# Country‐Level Burden Profiles for Fall‐Related Nursing Service Planning in Older Adults

**DOI:** 10.1155/jonm/4990192

**Published:** 2026-08-02

**Authors:** Lingling Xie, Hongshan Pu, Wenhua Jiang, Qian Chen, Ming Yang

**Affiliations:** ^1^ Center of Gerontology and Geriatrics, West China Hospital, Sichuan University, Chengdu, China, scu.edu.cn; ^2^ West China School of Nursing, Sichuan University, Chengdu, China, scu.edu.cn; ^3^ West China Fourth Hospital, Sichuan University, Chengdu, China, scu.edu.cn; ^4^ School of Public Health, Sichuan University, Chengdu, China, scu.edu.cn; ^5^ National Clinical Research Center for Geriatrics, West China Hospital, Sichuan University, Chengdu, China, scu.edu.cn; ^6^ Institute of Respiratory Health and Multimorbidity, West China Hospital, Sichuan University, Chengdu, China, scu.edu.cn

**Keywords:** falls, health services planning, long-term care, nursing services, older adults, rehabilitation

## Abstract

**Background:**

Falls are a source of disability, mortality, and loss of independence in later life, but evidence is usually presented as an epidemiologic burden rather than as comparative burden patterns that can inform nursing management and service planning.

**Methods:**

We conducted an ecological, age‐sex‐stratified study using GBD 2023 data for 204 countries and territories from 1990 to 2023. All countries and territories with the required GBD 2023 estimates were included. The analysis focused on adults aged 60 years or older to align with global older‐adult service‐planning conventions and the first older‐adult GBD age band. Main indicators were incidence rate, years lived with disability (YLD) rate, YLDs per 1000 falls, deaths per 100,000 falls, YLD share of disability‐adjusted life years (DALYs), and the share of YLDs contributed by adults aged 85 years or older. Four standardized domain scores were combined into an exploratory country‐level burden‐profile framework. Sensitivity analyses evaluated oldest‐old thresholds, percentile cutoffs, number/rate‐only reconstruction, sex‐specific models, and replacement of the prevention indicator with the age‐standardized incidence rate for falls.

**Results:**

In 2023, among adults aged 60 years or older, the global incidence rate of falls was 6600.3 per 100,000, the YLD rate was 1842.8 per 100,000%, and 69.5% of fall‐related DALYs were attributable to YLDs. The exploratory framework grouped countries into five country‐level burden profiles: 14 prevention‐intensive countries, 43 rehabilitation‐intensive countries, 43 mortality‐intensive countries, 14 long‐term‐care‐intensive countries, and 90 mixed‐profile countries, with the mixed profile being the largest group. Of the 90 mixed‐profile countries, 43 had multiple domains above threshold and 47 had no single domain above threshold. Across countries, incidence ranged from 417.7 to 27,003.8 per 100,000, YLD burden ranged from 152.6 to 795.2 YLDs per 1000 falls, deaths per 100,000 falls ranged from 335.4 to 6803.2, and the 85+ share of YLDs ranged from 1.58% to 28.53%. Women had higher incidence and YLD rates than men, whereas men had higher YLDs per 1000 falls and deaths per 100,000 falls. Sensitivity analyses showed partial but non‐uniform stability across specifications: 13 countries changed profile classification when the oldest‐old threshold was shifted from 85+ to 80+ years and 10 changed when the prevention domain was rebuilt using the age‐standardized incidence rate for falls, whereas 34 changed under the 80th‐percentile threshold and 73 under the number/rate‐only specification.

**Conclusions:**

Fall‐related burden in older adults showed distinct country‐level patterns across prevention, rehabilitation, mortality‐related burden, and long‐term support. Reorganizing burden structure beyond incidence alone may help describe cross‐national differences relevant to service planning. These ecological profiles summarize country‐level burden configurations that can support comparative nursing management review when interpreted alongside local workforce and service‐use data.

**Implication for Nursing Management:**

These ecological profiles may serve as starting points for local review when interpreted alongside measured workforce, infrastructure, and service‐use data. Prevention‐intensive profiles identify countries with comparatively high fall incidence; rehabilitation‐intensive profiles identify countries with comparatively high nonfatal postfall burden ratios; mortality‐intensive profiles identify countries with comparatively high fatal burden ratios; long‐term‐care‐intensive profiles identify countries in which fall‐related disability is more concentrated in adults aged 85 years or older; and mixed profiles identify either overlapping high‐burden domains or no single dominant domain.

## 1. Introduction

Falls are a major cause of injury‐related morbidity, mortality, and loss of independence in later life. The World Health Organization identifies falls as a leading source of injury burden worldwide, and previous Global Burden of Disease (GBD) analyses have shown that falls remain a substantial cause of death and disability across countries and regions [1, 2]. Classic epidemiologic work also established that falls in later life are common, multifactorial, and strongly related to functional vulnerability [3, 4]. As populations age, the clinical and public health consequences of falls increasingly extend beyond acute injury to persistent disability, functional decline, and institutional care needs, with substantial downstream costs for health systems [5–7].

Current prevention and management frameworks emphasize multifactorial risk reduction, exercise‐based interventions, and individualized assessment in older adults at increased risk of falling [8–10]. Earlier AGS/BGS guidance and later comparative evidence syntheses similarly support multidomain screening and structured prevention strategies [11–15]. Evidence also indicates that falls are shaped by multidimensional risk profiles that include age, prior falls, balance impairment, depression, visual problems, frailty, and cognitive vulnerability [16–21]. However, these intervention and risk‐factor frameworks are usually derived from clinical or community studies rather than from a global assessment of how fall burden is structured across health systems.

Existing international burden studies have largely described temporal and geographic patterns of falls without reorganizing those patterns into country‐level burden profiles potentially relevant to nursing management and service planning ([2, 22] Disease and Injury and Risk Factor Collaborators, 2025). For nursing management and health‐service planning, incidence alone is an incomplete comparative descriptor. Countries may have similar event rates but very different nonfatal burden per fall, fatal burden per fall, or oldest‐old burden concentration, indicating different burden configurations across prevention, rehabilitation, acute care, and long‐term support. To our knowledge, no prior global falls study has used multiple burden dimensions to summarize country‐level comparative profiles across these domains.

We therefore used GBD 2023 data to identify cross‐national country‐level burden profiles for fall‐related burden in adults aged 60 years or older, to quantify age‐sex gradients in postfall burden patterns, and to evaluate whether these country‐level comparisons were robust to alternative threshold and indicator specifications. Our aim was to generate an ecological burden‐structure framework for cross‐national comparison that could inform, but not replace, local service planning when interpreted alongside external data on workforce, infrastructure, and service use.

## 2. Methods

### 2.1. Study Design and Data Source

This was an ecological, cross‐national, age‐sex‐stratified study using publicly available data from the GBD Study 2023 [22] Results Tool and the accompanying GBD framework publications [22, 23]. The analysis used downloadable, aggregated outputs from the public IHME Results Tool rather than collaborator‐only internal materials. The unit of analysis was the country or territory. We examined fall‐related burden from 1990 to 2023 across 204 countries and territories. All 204 countries and territories available in [22] for the required indicators were included, and no additional country‐level inclusion or exclusion criteria were applied.

### 2.2. Population, Stratification, and Measures

No individual participants were recruited. Instead, we analyzed publicly available, deidentified, and aggregated country‐level estimates for adults aged 60 years or older. Age groups were 60–64, 65–69, 70–74, 75–79, 80–84, 85–89, 90–94, and 95+ years. Sex strata were female, male, and both sexes combined. For the main country‐level analyses, we extracted 2023 counts for incidence, deaths, years lived with disability (YLDs), years of life lost (YLLs), disability‐adjusted life years (DALYs), and population.

We selected 60 years as the lower age threshold because the study was designed for global older‐adult service planning, for which the 60+ convention is widely used in international ageing work, and because 60–64 years is the first older‐adult five‐year age band consistently available in the GBD older‐population extracts used here. This choice was intended to retain global comparability rather than to imply that 60 years is the only valid threshold for older‐adult falls research.

The main indicators were incidence rate in adults aged 60 years or older, YLD rate in adults aged 60 years or older, YLDs per 1000 incident falls, deaths per 100,000 incident falls, YLD share of DALYs, and the share of YLDs contributed by adults aged 85 years or older. Incidence rate and YLD rate were calculated as crude rates per 100,000 population aged 60 years or older. Crude rates were selected because the framework was intended to approximate current service workload within older populations rather than to isolate etiologic differences after age standardization. The 85+ share of YLDs was calculated by dividing YLD counts in the 85–89, 90–94, and 95+ year groups by total YLDs in adults aged 60 years or older. YLDs per 1000 incident falls and deaths per 100,000 incident falls were treated as ecological burden ratios derived from aggregated counts and were not interpreted as patient‐level severity measures. The female‐to‐male ratio of YLDs per 1000 incident falls was defined by dividing the female value by the male value. For supporting risk‐attributable analyses, we also extracted age‐standardized fall‐related YLD rates attributable to 4 prespecified risks available in the public GBD outputs: low bone mineral density, occupational injuries, smoking, and high alcohol use.

### 2.3. Construction of Country‐Level Burden Profiles

For the primary 2023 country‐level classification, we constructed four burden‐structure domains for comparative nursing‐service‐planning interpretation: fall frequency, nonfatal disability burden after falls, fatal burden after falls, and oldest‐old dependency pressure relevant to longer‐term support. The prevention domain was based on the country‐specific incidence rate in adults aged 60 years or older. The rehabilitation domain was defined as the mean of the z‐standardized YLDs per 1000 incident falls and the z‐standardized YLD share of DALYs. The mortality domain was defined as the mean of the z‐standardized deaths per 100,000 incident falls and the z‐standardized YLL share of DALYs. The long‐term‐care domain was based on the z‐standardized 85+ share of YLDs. The YLD‐share and YLL‐share components were included to describe whether fall burden within total DALYs was more disability‐dominant or mortality‐dominant; because these 2 shares partition total DALYs, they were treated as complementary compositional markers rather than as independent validation signals or separate latent constructs.

For each country, we first calculated the raw indicators, then standardized each component indicator across the full set of 204 included countries using z scores. The rehabilitation and mortality domain scores were the arithmetic mean of their 2 standardized component indicators. Equal weighting was used because each indicator pair was intended to capture complementary aspects of the same domain and we had no external empirical basis for assigning differential weights. We then identified the 75th percentile of each domain‐score distribution, flagged countries exceeding each domain‐specific threshold, and applied a deterministic single‐domain assignment rule.

Domain‐specific high values were defined using the 75th percentile of the country distribution for each domain score. We selected this threshold as a pragmatic prespecified cut point to identify comparatively high‐burden countries while retaining sufficient countries in each profile for descriptive cross‐national comparison. Countries were classified as prevention‐intensive, rehabilitation‐intensive, mortality‐intensive, or long‐term‐care‐intensive when one and only one domain exceeded its threshold and that domain also had the highest score for that country. Countries with multiple high domains or with no single domain meeting the classification rule were assigned to the mixed profile. For descriptive interpretation, mixed‐profile countries were further labeled as multi‐high mixed when 2 or more domains exceeded threshold and as diffuse/no‐dominant mixed when no domain exceeded threshold. Incidence was used as an author‐defined proxy for the volume of fall events entering prevention and follow‐up pathways; YLDs per 1000 incident falls and YLD share of DALYs as author‐defined proxies for nonfatal postfall burden; deaths per 100,000 incident falls and YLL share of DALYs as author‐defined proxies for fatal burden; and the 85+ share of YLDs as an author‐defined proxy for concentration of fall‐related disability in the oldest‐old. The framework was designed as a comparative burden‐structure heuristic potentially relevant to service planning rather than as a direct measure of unmet nursing care, observed service demand, or an individual‐level risk score. We did not treat the profile labels as latent constructs or perform formal construct‐validation testing. We also acknowledge that alternative cut points or unsupervised approaches, such as cluster analysis, k‐means, or latent profile analysis, could yield different profile structures. Official GBD sociodemographic index (SDI) quintiles were merged for stratified summaries.

### 2.4. Supplementary and Sensitivity Analyses

Supplementary analyses included global background trends from 1990 to 2023, country rankings of the core indicators in 2023, and country‐level indicator correlation matrices. Sensitivity analyses evaluated 5 alternative specifications: replacing the prevention‐domain indicator with the GBD age‐standardized incidence rate for falls, replacing the oldest‐old threshold from 85+ to 80+ years, raising the domain threshold from the 75th to the 80th percentile, reconstructing the priority profiles using number/rate‐only indicators, and fitting sex‐specific profile models. Country‐level estimated annual percentage changes (EAPCs) were also calculated for incidence rate, YLDs per 1000 falls, deaths per 100,000 falls, and the 85+ share of YLDs.

### 2.5. Statistical Analysis

Analyses were descriptive and exploratory. Country‐level correlations were assessed using Spearman rank correlation. EAPCs were estimated using log‐linear regression across annual country‐specific values from 1990 to 2023. The indicator set was chosen to reflect burden frequency, event‐normalized burden ratios, and disability structure at the health‐system level rather than to reproduce trial outcome sets, complementing prior falls outcome standardization work [24]. Because several main indicators were derived from aggregated counts rather than supplied as native GBD uncertainty estimates, no formal hypothesis testing or uncertainty intervals were presented for those derived ratios. Profile assignment was therefore deterministic and based on point estimates only. We did not propagate uncertainty from the underlying GBD estimates into the derived ratios, domain scores, or final profile assignments, and we could not estimate country‐specific probabilities of profile membership or quantify how often countries might change profile under full uncertainty propagation alone. The country‐level correlation matrix was used to summarize covariation among the selected indicators; mathematically complementary DALY‐share indicators were not treated as construct‐validation evidence. The public download files used here also do not provide a simple country‐by‐country audit of how much each released estimate depends on direct observation versus modeled borrowing for the specific derived indicators analyzed in this study.

Analyses and figures were produced in R Version 4.4.2 using the major packages data.table, dplyr, readr, ggplot2, patchwork, sf, and scales.

### 2.6. Ethics Statement

This study used publicly available, deidentified, and aggregated data and did not involve direct contact with human participants. Ethics approval and informed consent were therefore not required.

## 3. Results

### 3.1. Overview of Comparative Burden Indicators and Profile Groups in 2023

In 2023, the global incidence rate of falls among adults aged 60 years or older was 6600.3 per 100,000, with a YLD rate of 1842.8 per 100,000 and 279.2 YLDs per 1000 falls; 69.5% of total fall‐related DALYs were attributable to YLDs, and 13.0% of YLDs were contributed by adults aged 85 years or older (Table 1). Female older adults had a higher incidence rate and YLD rate than males (7840.7 vs 5168.4 per 100,000 and 1881.9 vs 1797.6 per 100,000, respectively), whereas males had higher YLDs per 1000 falls and higher deaths per 100,000 falls (347.8 vs 240.0 and 1080.7 vs 678.9, respectively) (Table 1). Across SDI strata, High SDI settings had the highest observed incidence and YLD rates, whereas low SDI settings had the highest observed deaths per 100,000 falls and the lowest YLD share of DALYs, descriptively indicating a shift from disability‐dominant to mortality‐dominant fall burden with decreasing development level (Table 1).

**TABLE 1:** Key nursing‐priority indicators for fall‐related burden in 2023 by global, SDI, and sex strata.

Stratum	Sex	Incidence rate	YLD rate	YLDs per 1000 falls	Deaths per 100,000 falls	YLD share of DALYs (%)	85+ share of YLDs (%)
Global	Both	6600.3	1842.8	279.2	824.9	69.5	13.0
Global	Female	7840.7	1881.9	240.0	678.9	72.1	16.9
Global	Male	5168.4	1797.6	347.8	1080.7	66.7	8.3
High SDI	Both	13,975.8	2961.1	211.9	503.1	77.0	17.8
High SDI	Female	16,622.6	3304.3	198.8	405.3	81.0	21.4
High SDI	Male	10,854.5	2556.3	235.5	679.8	71.6	12.3
High‐middle SDI	Both	5490.9	1990.0	362.4	645.5	79.8	10.4
High‐middle SDI	Female	6065.2	1778.2	293.2	539.0	81.4	14.5
High‐middle SDI	Male	4816.5	2238.9	464.8	803.1	78.4	6.6
Middle SDI	Both	3429.5	1089.9	317.8	1139.8	63.6	12.1
Middle SDI	Female	4022.0	1049.8	261.0	920.5	66.9	16.8
Middle SDI	Male	2724.4	1137.7	417.6	1525.0	60.4	6.9
Low‐middle SDI	Both	4089.2	1181.9	289.0	2009.6	46.1	9.1
Low‐middle SDI	Female	5593.1	1396.0	249.6	1582.1	49.3	11.3
Low‐middle SDI	Male	2465.9	950.8	385.6	3056.3	41.7	5.7
Low SDI	Both	1727.9	517.8	299.7	3796.3	32.8	12.8
Low SDI	Female	2032.2	560.0	275.6	2894.4	39.2	16.4
Low SDI	Male	1395.2	471.6	338.0	5232.2	27.1	8.1

*Note:* Incidence rate and YLD rate are crude rates per 100,000 adults aged 60 years or older in 2023. YLDs per 1000 falls, deaths per 100,000 falls, YLD share of DALYs, and 85+ share of YLDs are descriptive structural indicators derived from aggregated country‐level counts; no uncertainty intervals are shown.

Abbreviations: DALYs, disability‐adjusted life years; SDI, sociodemographic index; YLDs, years lived with disability.

The country‐level burden‐profile framework grouped 204 countries into five categories: 14 prevention‐intensive, 43 rehabilitation‐intensive, 43 mortality‐intensive, 14 long‐term‐care‐intensive, and 90 mixed‐profile countries, making the mixed profile the largest single category (Table 2). Prevention‐intensive profiles were defined by dominant prevention scores above the 75th percentile threshold, rehabilitation‐intensive profiles by high YLDs per 1000 falls and high YLD share of DALYs, mortality‐intensive profiles by high deaths per 100,000 falls and high YLL share of DALYs, and long‐term‐care‐intensive profiles by a high 85+ share of YLDs (Table 2). Example countries included Greenland, the United States of America, and Hungary for the prevention‐intensive group; Cook Islands, the United Arab Emirates, and Turkmenistan for the rehabilitation‐intensive group; Chad, Madagascar, and South Sudan for the mortality‐intensive group; and Comoros, Cabo Verde, and Uruguay for the long‐term‐care‐intensive group (Table 2). Among the 90 mixed‐profile countries, 43 had multiple domains above threshold and 47 had no domain above threshold, indicating that the mixed group captured both overlapping high‐burden configurations and diffuse no‐dominant patterns. The complete country‐by‐country classification, including mixed‐profile subtype labels, is provided in Table S3.

**TABLE 2:** Operational definitions and 2023 distribution of nursing priority profiles.

Profile	Operational definition	Dominant indicator set	Threshold rule	Country count	Representative countries
Prevention‐intensive	Highest dominant score in prevention domain and above the 75th percentile threshold	High incidence rate in adults aged 60+	Prevention score ≥ 0.084	14	Greenland; United States of America; Hungary
Rehabilitation‐intensive	Highest dominant score in rehabilitation domain and above the 75th percentile threshold	High YLDs per 1000 falls and high YLD share of DALYs	Rehabilitation score ≥ 0.491	43	Cook Islands; United Arab Emirates; Turkmenistan
Mortality‐intensive	Highest dominant score in mortality domain and above the 75th percentile threshold	High deaths per 100,000 falls and high YLL share of DALYs	Mortality score ≥ 0.575	43	Chad; Madagascar; South Sudan
Long‐term‐care‐intensive	Highest dominant score in long‐term‐care domain and above the 75th percentile threshold	High 85+ share of YLDs	Long‐term care score ≥ 0.608	14	Comoros; Cabo Verde; Uruguay
Mixed	Multiple domains high or no single domain exceeding the threshold	No single dominant pathway	Applied when the single‐domain rule was not met	90	Andorra; Japan; Slovenia

*Note:* Thresholds were defined as the 75th percentile of the standardized domain score distribution across 204 countries. Representative countries are the top three countries within each profile according to the dominant domain score. Mixed indicates countries with either multiple domains above threshold or no single domain that met the profile rule.

Abbreviations: DALYs, disability‐adjusted life years; YLDs, years lived with disability; YLLs, years of life lost.

### 3.2. Cross‐National Heterogeneity in Country‐Level Burden Profiles

**FIGURE 1:**
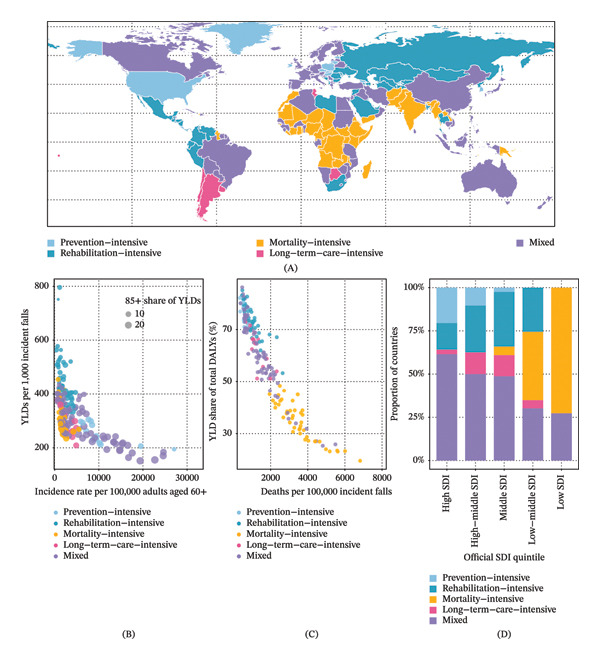
Global distribution of country‐level burden profiles for fall‐related burden in older adults in 2023. (A) The geographic distribution of five country‐level burden profiles. (B) Country‐level incidence rate in adults aged 60 years or older versus YLDs per 1000 incident falls, with point size indicating the percentage of YLDs contributed by adults aged 85 years or older. (C) Deaths per 100,000 incident falls versus the proportion of DALYs attributable to YLDs. (D) The distribution of profiles across SDI quintiles. These profile assignments are country‐level ecological planning summaries derived from modeled burden estimates and are not individual‐level risk categories. YLDs, years lived with disability; DALYs, disability‐adjusted life years; SDI, sociodemographic index.

Marked between‐country heterogeneity was observed in the distribution of country‐level burden profiles (Figure 1). All 204 countries were mapped to a profile, with only Tokelau requiring manual point placement on the map (Figure 1A). Across countries, incidence rates ranged from 417.7 to 27,003.8 per 100,000 adults aged 60 years or older, YLD burden ranged from 152.6 to 795.2 YLDs per 1000 falls, deaths per 100,000 falls ranged from 335.4 to 6803.2, and the YLD share of DALYs ranged from 19.6% to 86.1% (Figure 1B,C). The world map and SDI‐stratified distribution showed descriptive geographic clustering of profiles, with mixed profiles remaining the most common pattern across all SDI strata, whereas mortality‐intensive profiles appeared more common in lower‐SDI settings and long‐term‐care‐intensive profiles appeared more common in more developed settings (Figure 1A,D). The joint distributions in Figure 1B,C further indicated that incidence burden, nonfatal burden per fall, deaths per fall‐related event burden, and disability‐dominant burden structure varied substantially across countries rather than aligning on a single linear gradient.

### 3.3. Comparison of Burden‐Structure Domains Across Profiles

**FIGURE 2:**
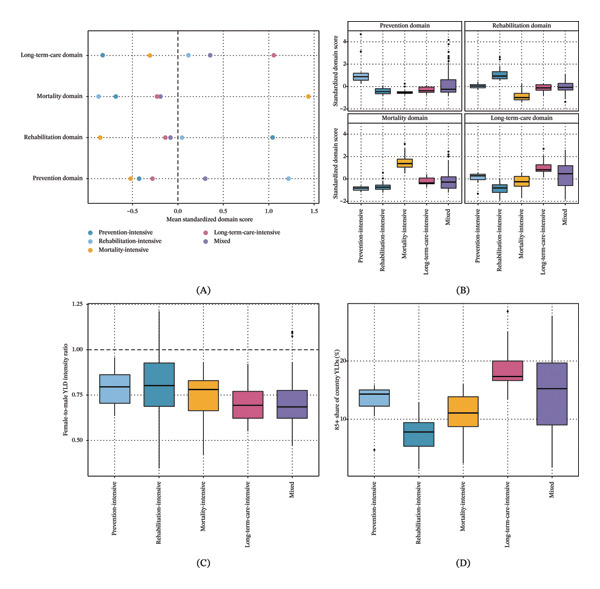
Country‐level burden domains across profile groups. (A) Mean standardized scores for the prevention, rehabilitation, mortality, and long‐term‐care domains across the five profiles. (B) The distribution of standardized domain scores within each profile. (C) The female‐to‐male ratio of YLDs per 1000 incident falls across profiles. (D) The percentage of country‐level YLDs contributed by adults aged 85 years or older across profiles. These profile assignments are country‐level ecological planning summaries derived from modeled burden estimates and are not individual‐level risk categories. YLDs, years lived with disability.

The domain‐based comparison showed that each profile was characterized by a different burden‐structure configuration (Figure 2). Prevention‐intensive countries showed the highest standardized prevention‐domain scores, rehabilitation‐intensive countries showed the highest observed rehabilitation‐domain scores, mortality‐intensive countries showed the highest observed mortality‐domain scores, and long‐term‐care‐intensive countries showed the highest observed long‐term‐care scores (Figure 2A,B). These differences translated into measurable contrasts in sex gaps and oldest‐old care pressure. The female‐to‐male ratio of YLDs per 1000 falls ratio ranged from 0.35 to 1.21 across countries, but the distribution differed across profiles, indicating that sex‐specific postfall burden patterns were not uniform across health‐system contexts (Figure 2C). Similarly, the proportion of country‐level YLDs contributed by adults aged 85 years or older ranged from 1.58% to 28.53%, with the highest levels concentrated in the long‐term‐care‐intensive group (Figure 2D). Together, these findings show that the five profiles occupied different positions across the standardized domains, sex‐gap distributions, and oldest‐old burden metrics (Figure 2), but they do not in themselves measure staffing, service capacity, or unmet care.

### 3.4. Age‐Sex Variation in Postfall Burden Patterns

**FIGURE 3:**
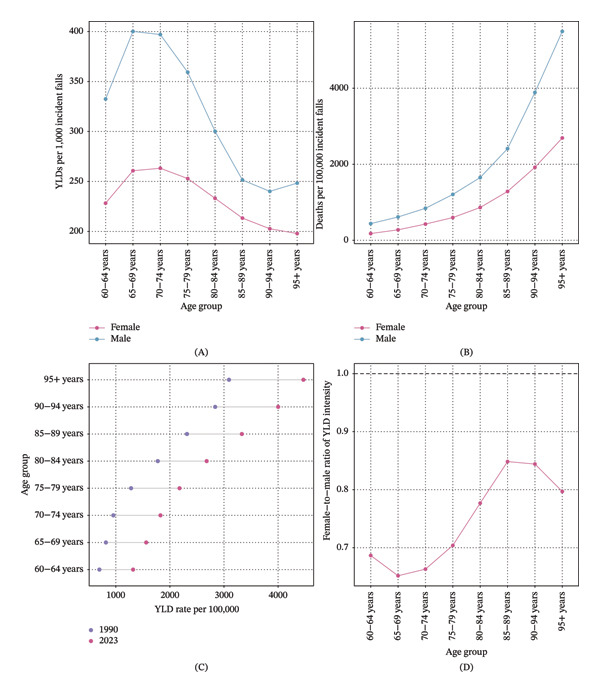
Age‐sex gradients in postfall burden patterns. (A) Age‐specific YLDs per 1000 incident falls in females and males in 2023. (B) Age‐specific deaths per 100,000 incident falls in females and males in 2023. (C) Age‐specific YLD rates in 1990 and 2023. (D) The female‐to‐male ratio of YLDs per 1000 incident falls across age groups in 2023. YLDs, years lived with disability.

Age‐ and sex‐specific analyses showed that incidence, YLD burden per fall, and deaths per fall did not follow the same pattern (Figure 3). Across age groups, YLDs per 1000 falls increased from ages 60–64 years to early old age and then declined at older ages, reaching 197.9 in females and 248.3 in males at ages 95 years or older (Figure 3A). Deaths per 100,000 falls rose sharply with age, reaching 2692.5 in females and 5496.0 in males at ages 95 years or older (Figure 3B). Age‐specific YLD rates also shifted upward over time; for example, among adults aged 85–89 years, the YLD rate increased from 2315.9 per 100,000 in 1990 to 3330.7 per 100,000 in 2023 (Figure 3C). Across all age groups, the female‐to‐male ratio of YLDs per 1000 falls remained below 1.0, ranging from 0.65 to 0.85, indicating consistently higher YLD burden per fall in males despite higher overall fall incidence in females (Figure 3D).

### 3.5. Supplementary Analyses and Robustness Checks

The broader temporal context is shown in Figure S3. From 1990 to 2023, global fall‐related burden in older adults increasingly shifted toward disability‐dominant burden, with YLDs accounting for 69.5% of DALYs in 2023; adults aged 85 years or older contributed 13.0% of YLDs and 16.6% of DALYs in that year (Figure S1). Country rankings for the four core comparative burden indicators are summarized in Figure S2: Greenland, Canada, the Netherlands, Slovenia, and the United States of America ranked highest for incidence; Cook Islands, the United Arab Emirates, Turkmenistan, Kiribati, and Qatar ranked highest for YLDs per 1000 falls; Chad, Madagascar, South Sudan, Ethiopia, and Guinea ranked highest for deaths per 100,000 falls; and Comoros, Andorra, Japan, Slovenia, and Cuba ranked highest for the 85+ share of YLDs (Figure S2).

Sensitivity analyses showed that the burden‐profile framework was directionally stable but not invariant to alternative specifications. When the oldest‐old threshold was shifted from 85+ to 80+, only 13 countries changed profile, 191 of 204 countries remained on the diagonal of the transition matrix, and the country‐level 80+ and 85+ YLD shares remained highly concordant (Spearman rho = 0.986) (Figure S3). When the prevention domain was reconstructed using the GBD age‐standardized incidence rate for falls, 10 countries changed profile classification, and 194 of 204 retained their primary profile assignment (Table S3). When the domain threshold was raised from the 75th to the 80th percentile, 34 countries changed classification, and diagonal stability decreased to 170 countries, with the mixed group expanding from 90 to 108 countries (Figure S4). When the long‐term‐care domain was reconstructed using number/rate‐only indicators, reclassification became more substantial: 73 countries changed groups, diagonal stability decreased to 131 countries, and the mixed group increased to 120 countries (Figure S5). Sex‐specific modeling also showed substantial, but incomplete, stability of the framework: agreement with the both‐sex classification was 85.3% for the female model and 80.4% for the male model (Figure S6).

Risk‐attributable analyses across the 4 prespecified risks, namely, low bone mineral density, occupational injuries, smoking, and high alcohol use, showed that low bone mineral density was the largest contributor in the both‐sex aggregate and among females, whereas occupational injuries were higher among males in the global and SDI‐stratified comparisons (Figure S7; Table S6). Finally, the country‐level indicator correlation matrix summarized covariation among the selected indicators: deaths per 100,000 falls was strongly positively correlated with the YLL share of DALYs (rho = 0.946), whereas the YLD share and YLL share of DALYs were perfectly inversely correlated because they partition total DALYs (rho = −1.000) (Figure S8).

Detailed country‐level and age‐stratified values are provided in the supporting tables. Table S1 presents the raw 2023 country‐level indicators for all 204 countries. Table S2 summarizes country‐specific EAPCs from 1990 to 2023 and shows wide heterogeneity in incidence trends, with incidence EAPCs ranging from −2.198 to 4.335. Table S3 provides the full primary and sensitivity‐based profile assignments for all countries, including the age‐standardized incidence sensitivity analysis. Table S4 gives age‐ and sex‐stratified indicators underlying the main age‐sex analyses. Table S5 summarizes oldest‐old pressure indicators, showing that globally 24.6% of YLDs and 29.0% of DALYs were contributed by adults aged 80 years or older, with the corresponding 85+ shares being 13.0% and 16.6%, respectively. Table S6 provides the detailed sex‐ and SDI‐specific risk‐attributable YLD rates for the 4 prespecified risk factors.

## 4. Discussion

In this ecological analysis of 204 countries and territories, fall‐related burden in older adults clustered into distinct country‐level burden profiles. These profiles summarized different combinations of prevention burden, nonfatal postfall burden, fatal burden, and oldest‐old dependency pressure that may be relevant to nursing management review and service planning. The main contribution was the reorganization of existing burden indicators into a comparative planning framework that highlighted marked cross‐national heterogeneity, concentration of oldest‐old care pressure in a subset of countries, and divergence between YLD burden per fall and deaths per fall across age and sex strata.

These findings extend previous GBD work on falls. Earlier global estimates showed that falls represent a substantial source of morbidity and mortality worldwide, but they did not frame the burden as a nursing management‐oriented comparative planning problem [2]. Using the updated [22] platform and a nursing‐oriented classification strategy, the present study shows that countries with apparently similar fall burden can occupy different country‐level profiles because incidence, nonfatal burden per fall, deaths per fall, and oldest‐old dependency do not move together. This distinction may be relevant when service leaders compare countries, because it shows that burden structure varies beyond event frequency alone. Our incremental contribution beyond prior GBD studies is to reorganize routinely reported burden indicators into a comparative service‐planning framework spanning prevention burden, nonfatal postfall burden, fatal burden, and oldest‐old dependency across countries.

The four domains were selected to represent event volume, nonfatal postfall burden, fatal burden, and oldest‐old dependency pressure. Prevention was intended to reflect event frequency and front‐end workload; rehabilitation to reflect nonfatal disability burden after falls; mortality to reflect fatal burden and loss‐of‐life burden after falls; and long‐term care to reflect the concentration of disability in the oldest‐old population, where dependency and continuity needs are often greatest. These dimensions map onto recognizable nursing management questions, although they were not calibrated against staffing levels, service use, unmet care, or patient‐level outcomes. The YLD‐share and YLL‐share components were included to distinguish disability‐dominant from mortality‐dominant burden composition within total DALYs, not to serve as independent validation markers. The framework therefore provides a comparative structure for organizing country‐level burden patterns and would benefit from further validation against workforce, service‐use, and long‐term‐care data.

The mixed profile was the largest category and comprised 2 distinct patterns: countries with multiple domains above threshold and countries with no domain above threshold. This group highlights that single‐domain labels do not capture all cross‐national variation. Mixed profiles can therefore be read as descriptive markers of either overlapping high‐burden configurations or diffuse no‐dominant patterns, rather than as evidence of a single coherent service pathway.

The contrast between mortality‐intensive and long‐term‐care‐intensive settings is particularly relevant. Lower‐SDI countries more often clustered in the mortality‐intensive pattern, whereas more developed settings more often carried higher oldest‐old disability pressure and more disability‐dominant burden. This pattern is consistent with the idea that the consequences of falls differ across health systems and demographic structures. Where populations are older and survival after injury is more common, the burden may accumulate more heavily as prolonged disability, rehabilitation demand, and long‐term care. By contrast, where access to acute and postacute care is more constrained, a greater share of burden may remain concentrated in fatal outcomes. These ecological contrasts describe system‐level patterns that merit validation against external service‐capacity and demographic data.

The age‐sex findings also add clinically relevant detail. With advancing age, deaths per 100,000 falls rose sharply, whereas YLDs per 1000 falls increased into early old age and then declined at older ages; meanwhile, the oldest‐old contribution to YLDs was especially prominent in the long‐term‐care‐intensive group. This is consistent with prior evidence that injurious falls are associated with worse disability trajectories, higher rates of long‐term nursing home admission, and persistent functional loss after serious fall injuries [25, 26]. More recent evidence has also suggested that injurious falls may signal broader vulnerability, including subsequent cognitive decline or dementia diagnosis, further strengthening the rationale for postfall surveillance in older adults [27].

Low bone mineral density emerged as the dominant risk‐attributable contributor to fall‐related YLD burden in the both‐sex aggregate and among females, whereas occupational injuries were higher among males in the global and SDI‐stratified comparisons. This finding aligns with both earlier and updated GBD analyses showing that low bone mineral density is a major and modifiable contributor to fracture‐related disability from falls in older populations ([28] Low Bone Mineral Density Collaborators, 2025). It also fits with broader evidence that falls arise from multidimensional vulnerability, including musculoskeletal weakness, balance problems, cognitive frailty, depression, and prior falls [16, 19, 20]. This context supports interpreting fall‐related disability burden within a broader musculoskeletal and frailty framework rather than as an isolated event phenomenon.

At the country‐comparison level, these profiles may help structure nursing management review and further local service assessment. A prevention‐intensive profile identifies relatively high incidence burden; a rehabilitation‐intensive profile identifies comparatively high nonfatal burden ratios after falls; a mortality‐intensive profile identifies comparatively high fatal burden ratios; a long‐term‐care‐intensive profile identifies greater concentration of disability among the oldest‐old; and a mixed profile identifies either overlapping high‐burden domains or no single dominant domain. Whether any of these descriptive patterns correspond to staffing requirements, referral organization, or service gaps must be assessed with local data on workforce capacity, service utilization, infrastructure, financing, and cultural context. This framing is broadly consistent with contemporary guidelines that treat falls as multidimensional rather than single‐cause phenomena [8–10, 14].

This study has several strengths. It used a global dataset covering 204 countries and territories, explicitly incorporated age and sex structure, translated country patterns into interpretable country‐level burden profiles, and tested robustness across multiple sensitivity analyses. The study also has limitations. First, GBD estimates depend on heterogeneous underlying data sources and statistical modeling, and reliance on modeled extrapolation is likely greater in some settings, particularly locations with weaker surveillance systems. Country estimates and any global summaries should therefore be interpreted as modeled comparative estimates rather than directly observed surveillance totals. The public outputs used for this study also do not provide a simple country‐by‐country audit of how much each released estimate reflects direct observation versus modeled borrowing for the specific derived indicators examined here, so we could not quantify which profile assignments were most model‐dependent. Second, because the public GBD outputs used here provide point estimates for the released indicators rather than uncertainty‐propagated values for all derived ratios, we did not propagate uncertainty into the domain scores or final profile assignments. We therefore cannot estimate country‐specific probabilities of profile membership or judge how often classification would change under full uncertainty propagation, especially in more data‐sparse settings. Third, the analysis was ecological and based on aggregated country‐level estimates, so the results should not be interpreted as individual‐level risk relationships, bedside nursing priorities, or direct evidence for clinical triage. Fourth, because the primary country profiles relied on crude rates within the population aged 60 years or older, differences in internal age composition across countries may have contributed to some between‐country contrasts. However, only 10 countries were reclassified when the prevention domain was rebuilt using the GBD age‐standardized incidence rate for falls, suggesting that the main profile structure was not solely driven by crude age composition. The long‐term‐care domain, however, intentionally incorporated oldest‐old concentration and therefore also captured demographic service pressure. Fifth, the burden‐profile framework is an exploratory burden‐reorganization heuristic; we did not perform formal construct‐validation testing, and the framework does not directly measure unmet nursing care, nursing quality, workforce shortages, or causal pathways. Some domain components were also intentionally complementary rather than statistically independent, particularly the YLD‐share and YLL‐share indicators that partition total DALYs. Sixth, the domain thresholds, equal‐weighting choices, and indicator combinations were prespecified analytic decisions made for interpretability rather than learned from an external validation target, although the accompanying sensitivity analyses were designed to show how alternative specifications changed classification. Alternative unsupervised methods, including cluster analysis, k‐means, or latent profile analysis, could yield different profile structures. Accordingly, the framework is more suitable for hypothesis generation and comparative description than for direct performance evaluation or operational allocation of local nursing services.

Future work should assess whether similar profiles are observed within subnational settings, whether they remain stable over subsequent GBD rounds, whether uncertainty propagation materially alters profile assignment, and whether alternative unsupervised methods yield comparable structures. Formal construct validation against rehabilitation access, nursing workforce capacity, service use, and long‐term care infrastructure, together with external evaluation of the mixed‐profile subtypes, would also be needed before the framework could be treated as a validated planning tool.

## 5. Conclusion

In this cross‐national ecological analysis of [22] data, fall‐related burden in older adults clustered into five country‐level burden profiles spanning prevention, rehabilitation, mortality‐related burden, and long‐term support. The results suggest that comparing burden structure, not incidence alone, may offer a more informative descriptive basis for cross‐national service‐planning comparisons.

These profiles provide ecological, country‐level burden summaries and are most informative when interpreted alongside measured workforce, infrastructure, and utilization data.

## Author Contributions

Concept and design: Lingling Xie, Qian Chen, and Ming Yang.

Data curation: Lingling Xie and Hongshan Pu.

Formal analysis and visualization: Lingling Xie and Hongshan Pu.

Interpretation of findings: Lingling Xie, Hongshan Pu, Wenhua Jiang, Qian Chen, and Ming Yang.

Drafting of the manuscript: Lingling Xie and Hongshan Pu.

Critical revision of the manuscript for important intellectual content: Lingling Xie, Hongshan Pu, Wenhua Jiang, Qian Chen, and Ming Yang.

Supervision: Qian Chen and Ming Yang.

## Funding

This study was supported by Grant RHM25212 from 1·3·5 Project of the State Key Laboratory of Respiratory Health and Multimorbidity, West China Hospital, Sichuan University.

## Disclosure

All authors have approved the final version of the manuscript. The funder/sponsor had no role in the design and conduct of the study; collection, management, analysis, and interpretation of the data; preparation, review, or approval of the manuscript; or the decision to submit the manuscript for publication.

## Ethics Statement

Ethics approval and informed consent were not required because this study used publicly available, deidentified, and aggregated data.

## Conflicts of Interest

The authors declare no conflicts of interest.

## Supporting Information

Additional supporting information can be found online in the Supporting Information section.

## Supporting information


**Supporting Information 1** Supporting Appendix. This single PDF file contains Figures S1‐S8 and Tables S1‐S6, including global background trends, country rankings, sensitivity analyses, sex‐specific stability analyses, risk‐attributable YLD analyses, country‐level indicator correlations, raw country‐level indicators, annual percentage changes, full profile assignments, age‐ and sex‐stratified indicators, oldest‐old pressure indicators, and sex‐ and SDI‐specific risk‐attributable YLD rates.

**Supporting Information 2** Reporting Guideline Checklist STROBE.

## Data Availability

The GBD 2023 data used in this study are publicly available from the Institute for Health Metrics and Evaluation GBD Results Tool. Derived analytic files, country‐level profile assignments, and code supporting the findings are available from the corresponding authors on reasonable request.
