# Supplementary material for: Country‐Level Burden Profiles for Fall‐Related Nursing Service Planning in Older Adults
Source: J Nurs Manag. 2026 Aug 2;2026:4990192. doi: 10.1155/jonm/4990192 (PMC13430041; doi:10.1155/jonm/4990192)
Supplement: Supplementary file 1 — Supporting Information 1 Supporting Appendix. This single PDF file contains Figures S1‐S8 and Tables S1‐S6, including global background trends, country rankings, sensitivity analyses, sex‐specific stability analyses, risk‐attributable YLD analyses, country‐level indicator correlations, raw country‐level indicators, annual percentage changes, full profile assignments, age‐ and sex‐stratified indicators, oldest‐old pressure indicators, and sex‐ and SDI‐specific risk‐attributable YLD rates. [file JONM-2026-4990192-s002.pdf]

Nursing Priority Profiles in Older Adults: A Cross-National Study of Fall-Related Care Needs

Lingling Xie[1,4†]; Hongshan Pu[1†]; Wenhua Jiang[2,3]; Qian Chen[1,4*]; Ming Yang[1,5,6*]

1. Center of Gerontology and Geriatrics, West China Hospital, Sichuan University, Chengdu, China

2. West China Fourth Hospital, Sichuan University, Chengdu, China

3. School of Public Health, Sichuan University, Chengdu, China

4. West China School of Nursing, Sichuan University, Chengdu, China

5. National Clinical Research Center for Geriatrics, West China Hospital, Sichuan University, Chengdu, China

6. Institute of Respiratory Health and Multimorbidity, West China Hospital, Sichuan University, Chengdu, China

† These authors contributed equally to this work.

Corresponding authors

Prof. Qian Chen, Center of Gerontology and Geriatrics and West China School of Nursing, Sichuan University, No. 37 Guoxue Lane, Chengdu 610041, China. Email: chen_qian@scu.edu.cn

Prof. Ming Yang, Center of Gerontology and Geriatrics and National Clinical Research Center for Geriatrics, West China Hospital, Sichuan University, No. 37 Guoxue Lane, Chengdu 610041, China. Email: yangmier@gmail.com; yangmier@scu.edu.cn

Funding

This study was supported by Grant RHM25212 from 1·3·5 Project of the State Key Laboratory of Respiratory Health and Multimorbidity, West China Hospital, Sichuan University.

Acknowledgments

None.

Declaration of competing interests

The authors have no known competing financial interests or personal relationships that could have appeared to influence the work reported in this paper.

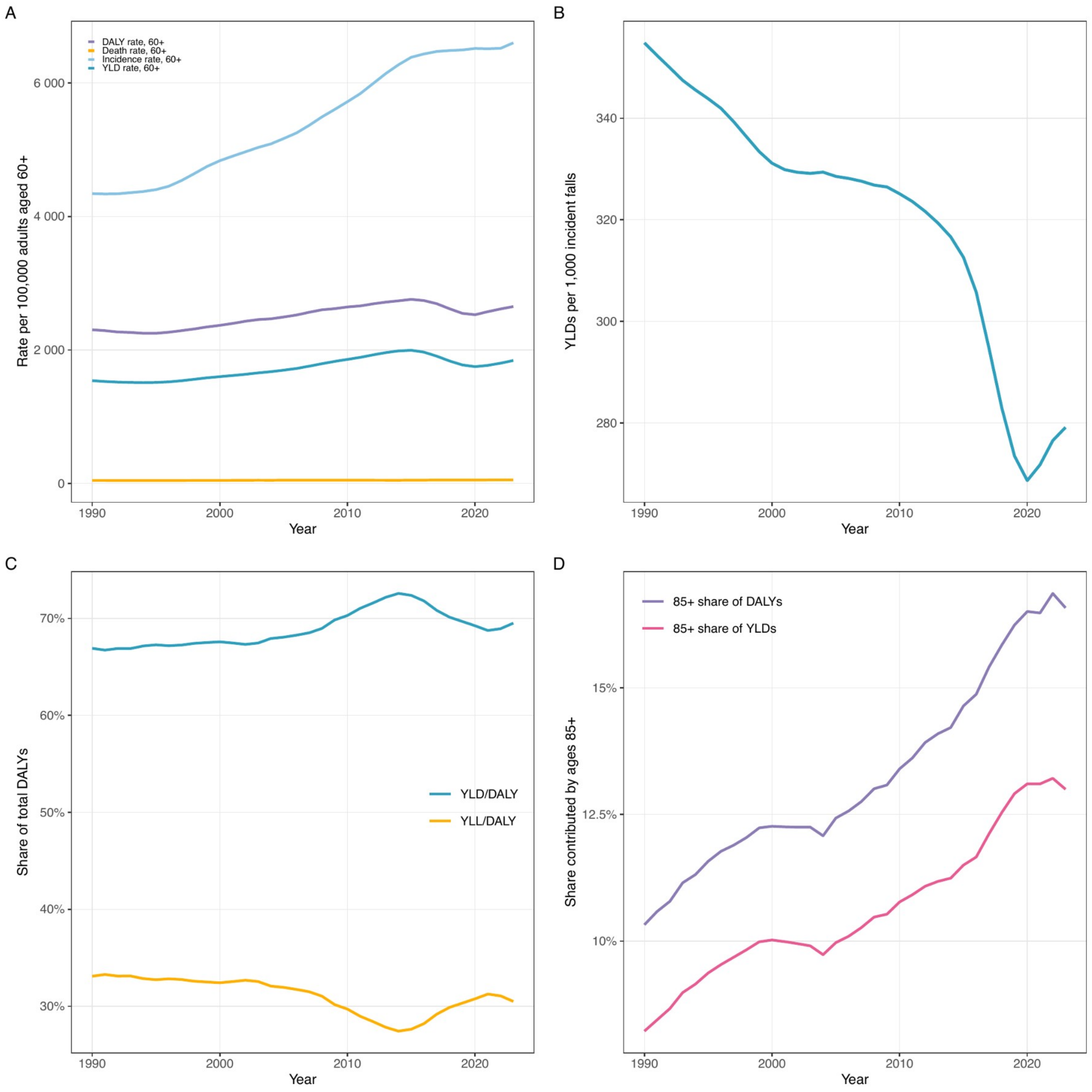

Figure S1. Global background trends in event burden and care burden, 1990-2023. Panel A shows global incidence, death, DALY, and YLD rates among adults aged 60 years or older from 1990 to 2023. Panel B shows global YLDs per 1,000 incident falls from 1990 to 2023. Panel C shows proportions of total DALYs attributable to YLDs and YLLs. Panel D shows the percentages of total YLDs and DALYs contributed by adults aged 85 years or older. DALYs, disability-adjusted life years; YLDs, years lived with disability; YLLs, years of life lost.

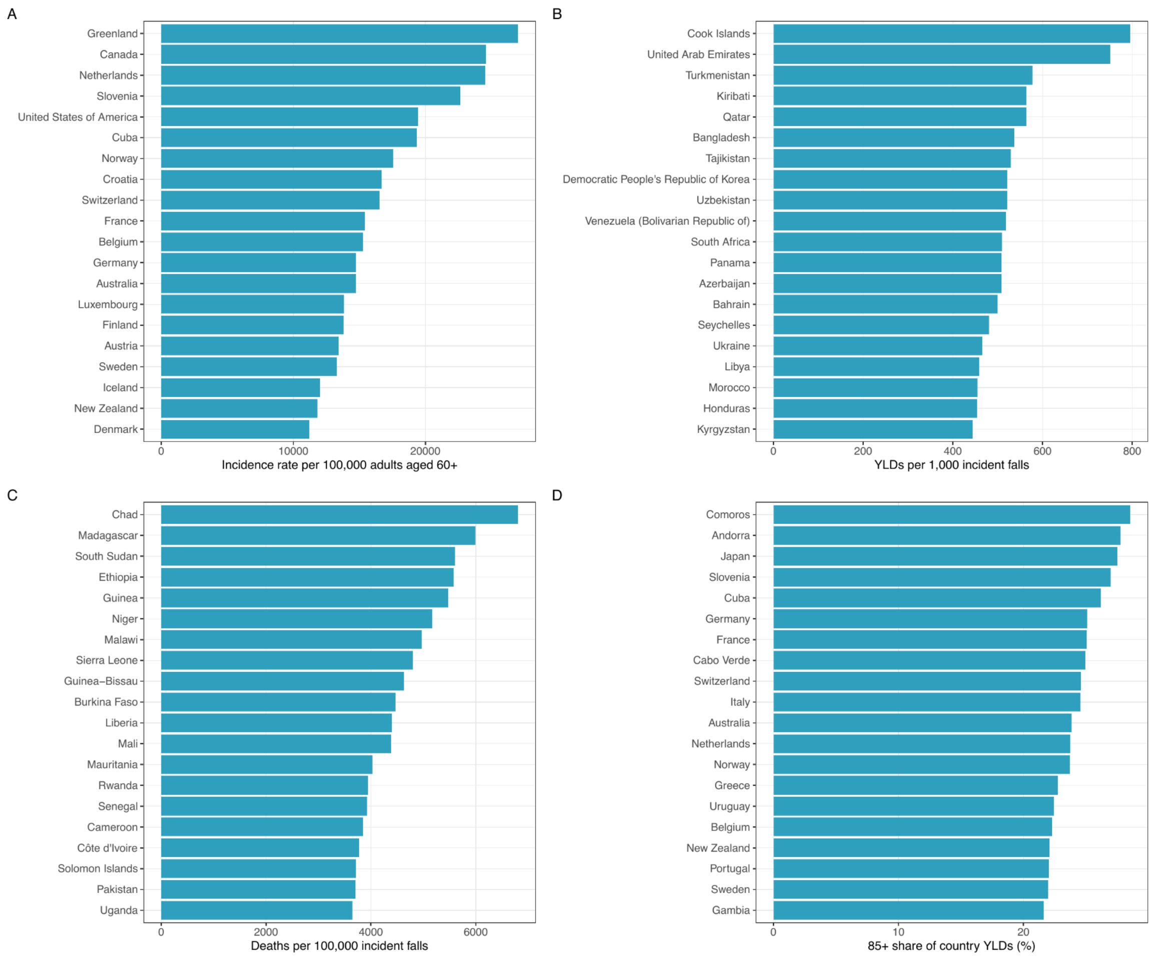

Figure S2. Top-ranked countries for the core nursing-priority indicators in 2023. Panels A-D show the top-ranked countries for incidence rate, YLDs per 1,000 incident falls, deaths per 100,000 incident falls, and the percentage of YLDs contributed by adults aged 85 years or older. YLDs, years lived with disability.

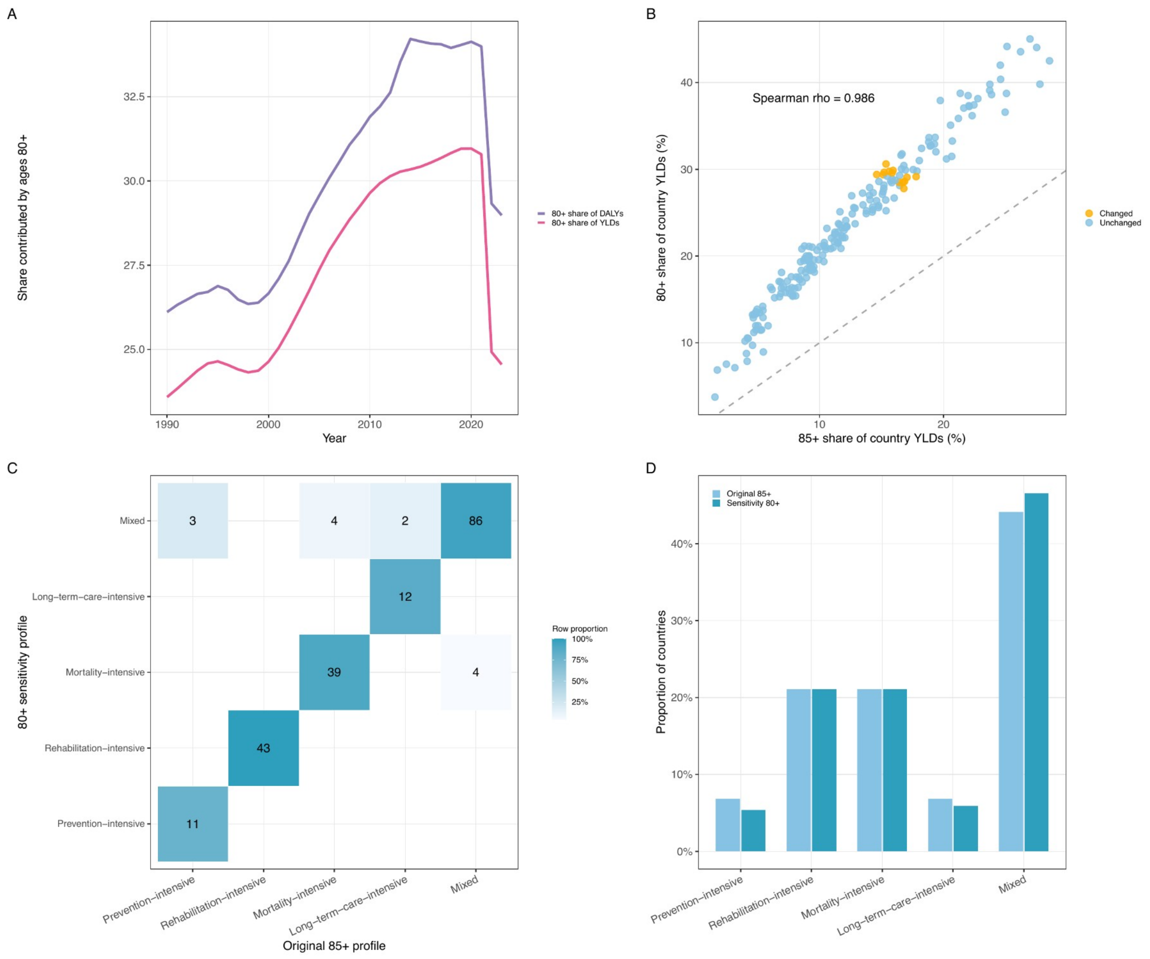

Figure S3. Sensitivity analysis using the 80-year threshold for oldest-old burden. Panel A shows global percentages of YLDs and DALYs contributed by adults aged 80 years or older. Panel B shows the country-level correlation between the 85+ and 80+ YLD shares in 2023. Panel C shows the transition matrix comparing the primary 85+ classification with the 80+ sensitivity classification. Panel D compares profile distributions under the 2 definitions. DALYs, disability-adjusted life years; YLDs, years lived with disability.

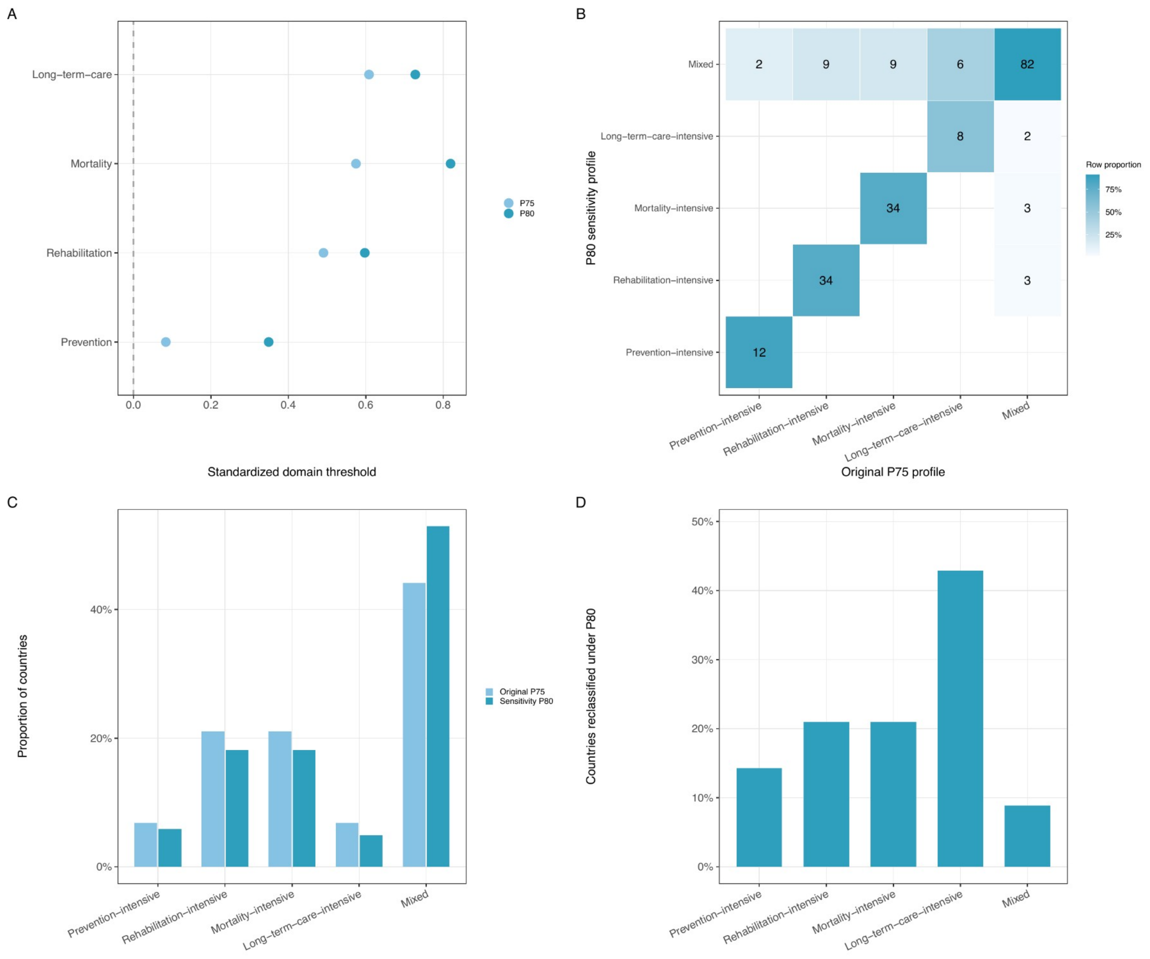

Figure S4. Sensitivity analysis using the 80th percentile threshold for profile classification. Panel A shows the domain thresholds under the primary 75th percentile rule and the sensitivity 80th percentile rule. Panel B shows the transition matrix comparing the 2 classifications. Panel C compares profile distributions under the 2 threshold rules. Panel D shows the proportion of countries reclassified under the 80th percentile rule.

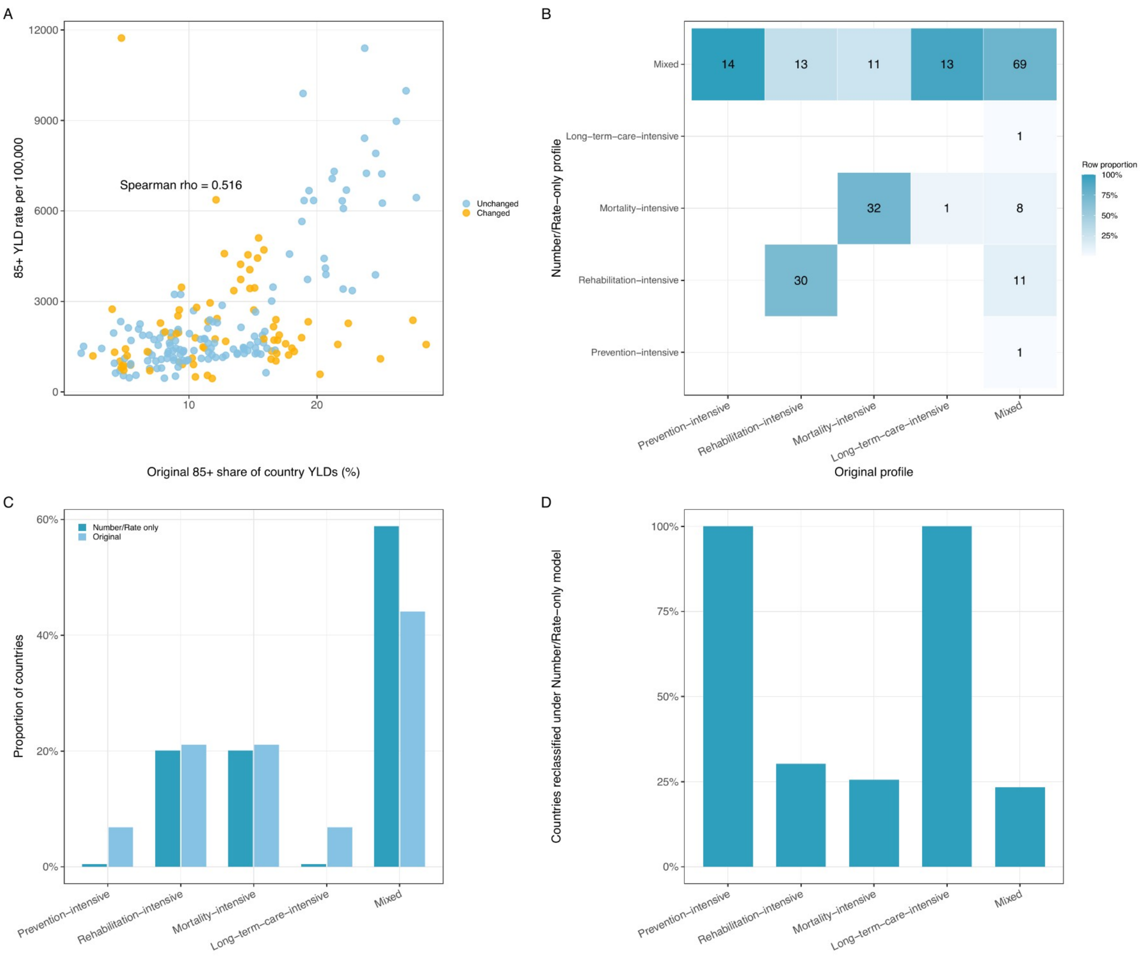

Figure S5. Sensitivity analysis using number/rate-only profile construction. Panel A shows the association between the primary 85+ YLD share indicator and the alternative 85+ YLD rate indicator. Panel B shows the transition matrix comparing the primary and number/rate-only profile classifications. Panel C compares profile distributions under the 2 specifications. Panel D shows the proportion of countries reclassified under the number/rate-only model. YLDs, years lived with disability.

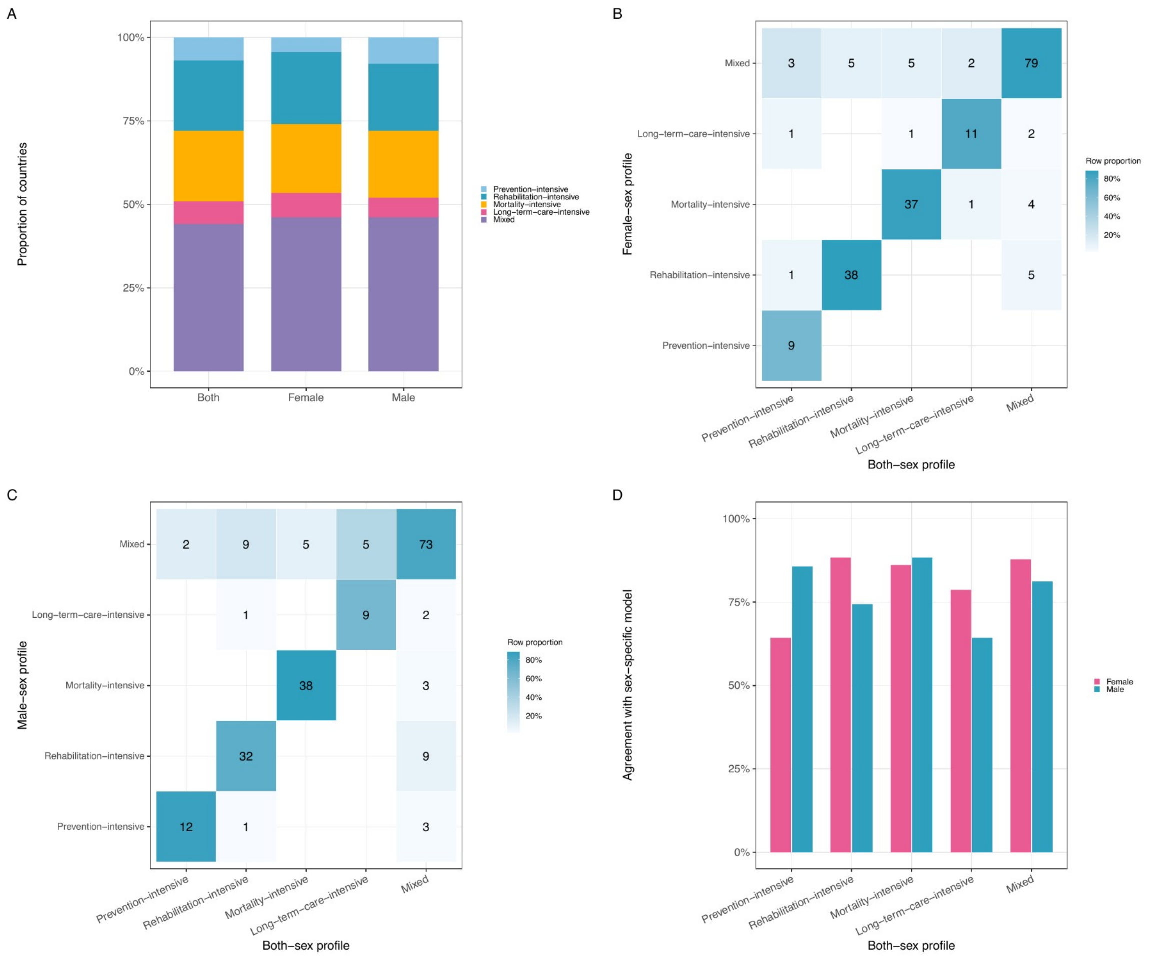

Figure S6. Sex-specific stability of nursing priority profiles. Panel A shows the distribution of profiles in the both-sex, female-only, and male-only models. Panels B and C show transition matrices comparing the sex-specific classifications with the both-sex classification. Panel D shows agreement between sex-specific and both-sex classifications across profiles.

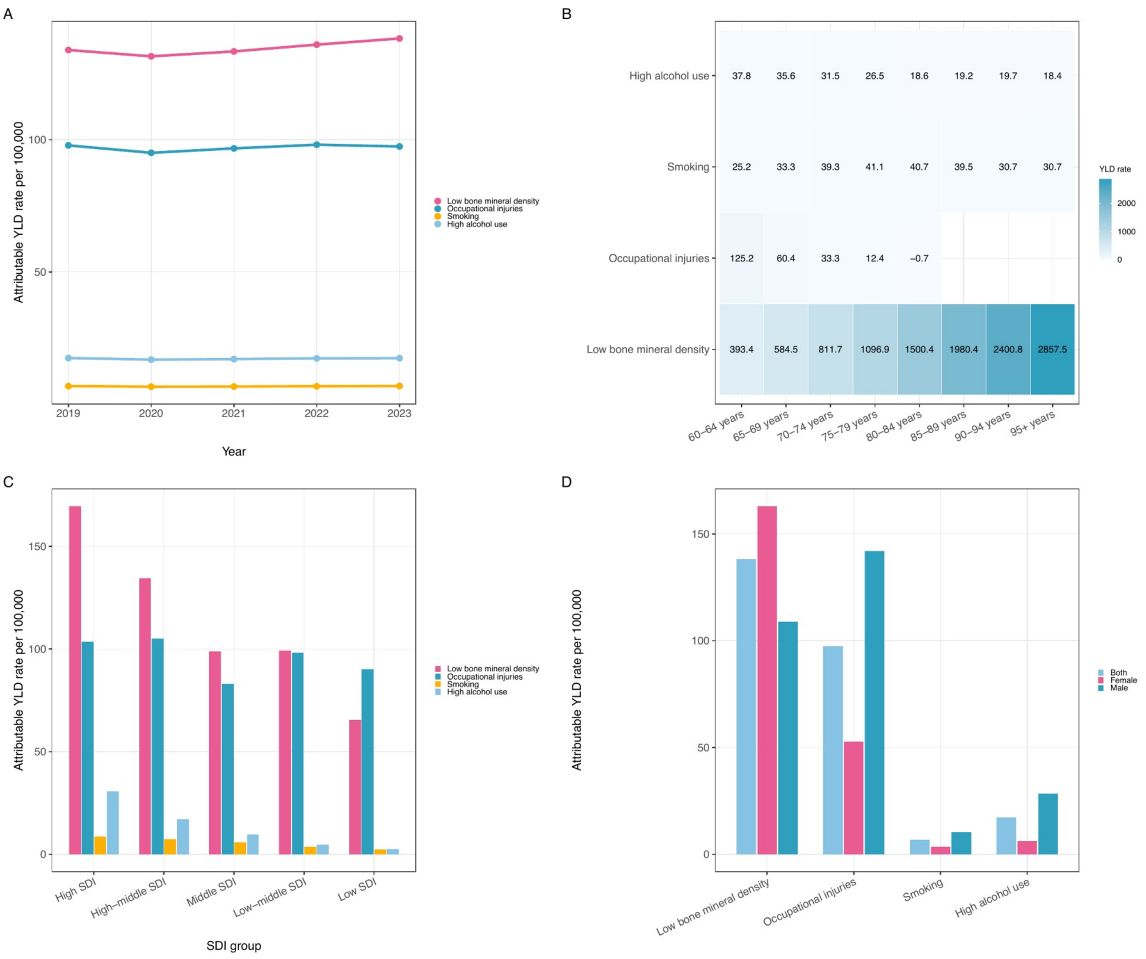

Figure S7. Risk-attributable YLD burden for falls in older adults. Panel A shows global age-standardized YLD rates attributable to selected risk factors from 2019 to 2023. Panel B shows age-specific attributable YLD rates in 2023. Panel C shows age-standardized attributable YLD rates by SDI group in 2023. Panel D shows age-standardized attributable YLD rates by sex in 2023. YLDs, years lived with disability; SDI, socio-demographic index.

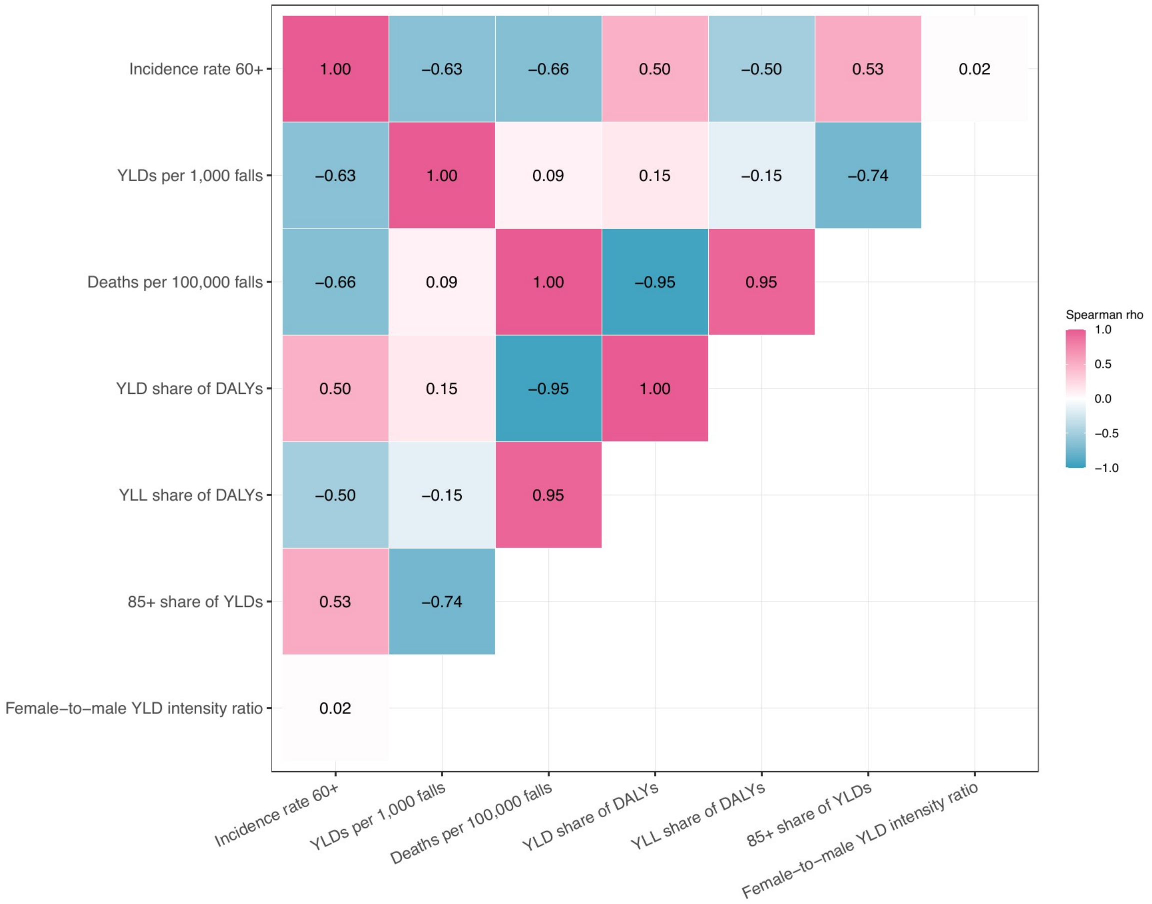

Figure S8. Country-level indicator correlation matrix. The matrix shows Spearman correlations among the main country-level indicators used in the nursing priority profile framework, including incidence rate in adults aged 60 years or older, YLDs per 1,000 incident falls, deaths per 100,000 incident falls, YLD share of DALYs, YLL share of DALYs, the percentage of YLDs contributed by adults aged 85 years or older, and the female-to-male YLD intensity ratio. DALYs, disability-adjusted life years; YLDs, years lived with disability; YLLs, years of life lost.

Table S1. Country-level raw indicators in 2023

Country	SDI quintile	Archetype	Incidence rate	YLDs per 1,000 falls	Deaths per 100,000 falls	YLD share of DALYs (%)	YLL share of DALYs (%)	85+ share of YLDs (%)	Female-to-male YLD intensity ratio
Afghanistan	Low SDI	Mortality-intensive	1,577.1	345.6	2,723.2	46.5	53.5	8.8	0.61
Albania	Middle SDI	Rehabilitation-intensive	2,553.6	412.2	484.8	85.0	15.0	8.5	0.94
Algeria	Middle SDI	Mixed	1,411.8	393.8	1,642.7	59.8	40.2	10.2	0.60
American Samoa	High-middle SDI	Mixed	1,651.6	376.1	1,811.7	54.7	45.3	7.6	0.67
Andorra	High SDI	Mixed	11,002.5	243.1	799.1	73.2	26.8	27.8	0.47
Angola	Low-middle SDI	Mortality-intensive	1,676.9	283.6	2,253.9	41.6	58.4	11.2	0.55
Antigua and Barbuda	High-middle SDI	Mixed	1,790.3	340.9	1,046.4	69.8	30.2	11.8	0.77
Argentina	High-middle SDI	Long-term-care-intensive	2,177.9	324.9	1,120.8	68.8	31.2	16.5	0.67
Armenia	Middle SDI	Rehabilitation-intensive	1,700.5	366.1	592.0	78.4	21.6	10.4	0.62
Australia	High SDI	Mixed	14,726.8	192.2	581.4	74.3	25.7	23.9	0.83
Austria	High SDI	Mixed	13,428.7	221.3	649.9	73.4	26.6	19.7	0.70
Azerbaijan	Middle SDI	Rehabilitation-intensive	848.2	507.9	632.6	79.8	20.2	4.9	0.69
Bahamas	High-middle SDI	Mixed	2,334.6	295.2	1,359.1	60.0	40.0	11.2	0.57
Bahrain	High-middle SDI	Rehabilitation-intensive	909.6	499.6	1,049.6	72.6	27.4	4.2	0.84
Bangladesh	Low-middle SDI	Rehabilitation-intensive	1,363.2	536.9	2,644.3	53.3	46.7	5.2	0.87
Barbados	High-middle SDI	Long-term-care-intensive	2,581.0	267.7	1,086.3	66.2	33.8	16.9	0.55
Belarus	High-middle SDI	Mixed	5,444.5	378.6	539.5	78.0	22.0	7.8	0.93
Belgium	High SDI	Mixed	15,268.9	215.1	611.2	73.8	26.2	22.3	0.67
Belize	Low-middle SDI	Mixed	2,412.7	284.3	1,094.8	65.0	35.0	12.0	0.69
Benin	Low SDI	Mixed	1,917.5	260.4	2,961.6	35.7	64.3	18.0	0.82
Bermuda	High SDI	Rehabilitation-intensive	2,756.5	359.2	1,069.7	73.1	26.9	12.9	0.86
Bhutan	Low-middle SDI	Mortality-intensive	4,262.2	260.6	2,435.8	41.1	58.9	15.2	0.69
Bolivia (Plurinational State of)	Low-middle SDI	Mixed	3,045.0	345.8	1,524.7	54.7	45.3	7.7	0.57
Bosnia and Herzegovina	High-middle SDI	Rehabilitation-intensive	3,482.5	335.7	609.5	77.5	22.5	10.5	1.15
Botswana	Middle SDI	Long-term-care-intensive	738.6	376.1	1,794.7	58.8	41.2	20.2	0.69
Brazil	Middle SDI	Mixed	3,936.2	315.1	1,432.5	59.7	40.3	12.2	0.61
Brunei Darussalam	High-middle SDI	Mixed	3,048.1	333.5	663.6	74.1	25.9	6.4	0.75
Bulgaria	High-middle SDI	Rehabilitation-intensive	2,841.1	395.6	670.0	78.8	21.2	8.5	0.91

Table S1. Country-level raw indicators in 2023

Country	SDI quintile	Archetype	Incidence rate	YLDs per 1,000 falls	Deaths per 100,000 falls	YLD share of DALYs (%)	YLL share of DALYs (%)	85+ share of YLDs (%)	Female-to-male YLD intensity ratio
Burkina Faso	Low SDI	Mixed	2,242.2	249.9	4,469.1	27.1	72.9	18.8	0.80
Burundi	Low SDI	Mortality-intensive	1,882.0	282.3	3,570.2	32.0	68.0	11.7	0.93
Cabo Verde	Low-middle SDI	Long-term-care-intensive	1,660.7	276.5	1,990.8	51.2	48.8	25.0	0.92
Cambodia	Low-middle SDI	Mixed	4,010.8	293.3	1,583.2	53.4	46.6	10.4	0.59
Cameroon	Low-middle SDI	Mortality-intensive	1,831.8	258.9	3,853.3	27.7	72.3	10.4	0.78
Canada	High SDI	Mixed	24,588.8	184.6	383.5	80.0	20.0	18.9	0.83
Central African Republic	Low SDI	Mortality-intensive	1,028.8	332.0	3,198.8	36.9	63.1	7.5	0.55
Chad	Low SDI	Mortality-intensive	1,372.5	285.4	6,803.2	19.6	80.4	9.8	0.81
Chile	High-middle SDI	Long-term-care-intensive	4,911.5	274.2	763.7	72.7	27.3	15.1	0.62
China	High-middle SDI	Mixed	5,729.0	392.0	613.4	81.8	18.2	8.9	0.61
Colombia	Middle SDI	Rehabilitation-intensive	3,595.6	404.1	716.5	78.8	21.2	11.9	1.04
Comoros	Low-middle SDI	Long-term-care-intensive	2,434.4	262.9	1,591.2	55.7	44.3	28.5	0.86
Congo	Low-middle SDI	Mortality-intensive	1,367.3	299.1	2,858.1	37.7	62.3	11.5	0.48
Cook Islands	High-middle SDI	Rehabilitation-intensive	1,169.4	795.2	2,329.7	67.0	33.0	6.7	0.81
Costa Rica	Middle SDI	Prevention-intensive	6,363.8	293.4	827.6	72.4	27.6	14.8	0.70
Croatia	High-middle SDI	Mixed	16,704.0	164.5	567.2	69.1	30.9	21.3	0.69
Cuba	Middle SDI	Mixed	19,343.9	152.6	799.8	60.7	39.3	26.2	0.63
Cyprus	High SDI	Prevention-intensive	9,445.4	243.8	761.0	72.3	27.7	15.4	0.63
Czechia	High SDI	Prevention-intensive	10,178.7	218.7	560.1	73.7	26.3	14.6	0.84
Côte d'Ivoire	Low SDI	Mortality-intensive	1,466.5	287.2	3,776.1	31.3	68.7	12.9	0.79
Democratic People's Republic of Korea	Low-middle SDI	Rehabilitation-intensive	3,647.7	520.9	971.3	76.7	23.3	4.7	0.60
Democratic Republic of the Congo	Low SDI	Mortality-intensive	1,277.8	311.4	3,607.2	33.0	67.0	9.9	0.59
Denmark	High SDI	Mixed	11,221.5	234.6	611.6	75.7	24.3	18.8	0.64
Djibouti	Low-middle SDI	Mortality-intensive	1,945.1	268.3	2,120.4	42.7	57.3	11.1	0.86
Dominica	High-middle SDI	Long-term-care-intensive	2,266.7	280.7	1,178.5	64.6	35.4	18.2	0.79
Dominican Republic	Middle SDI	Mixed	2,249.0	287.7	1,972.3	52.2	47.8	15.8	0.69
Ecuador	Middle SDI	Rehabilitation-intensive	4,121.2	355.3	819.7	74.5	25.5	12.2	0.49

Table S1. Country-level raw indicators in 2023

Country	SDI quintile	Archetype	Incidence rate	YLDs per 1,000 falls	Deaths per 100,000 falls	YLD share of DALYs (%)	YLL share of DALYs (%)	85+ share of YLDs (%)	Female-to-male YLD intensity ratio
Egypt	Low-middle SDI	Mixed	937.3	424.3	1,641.6	59.3	40.7	4.7	0.60
El Salvador	Low-middle SDI	Rehabilitation-intensive	3,756.9	387.0	1,250.5	67.3	32.7	11.6	0.74
Equatorial Guinea	Middle SDI	Mortality-intensive	1,563.3	268.3	3,030.9	34.3	65.7	14.3	0.59
Eritrea	Low SDI	Mortality-intensive	2,031.7	274.2	3,163.2	35.9	64.1	13.5	0.82
Estonia	High SDI	Prevention-intensive	7,691.0	305.0	539.6	79.0	21.0	15.1	0.87
Eswatini	Low-middle SDI	Mixed	599.0	401.6	1,739.9	58.7	41.3	11.8	0.61
Ethiopia	Low SDI	Mortality-intensive	1,730.7	267.4	5,573.4	23.5	76.5	15.6	0.87
Fiji	Middle SDI	Mixed	1,094.4	385.9	2,012.7	52.9	47.1	6.8	0.76
Finland	High SDI	Mixed	13,783.8	242.4	653.0	74.3	25.7	19.0	0.76
France	High SDI	Mixed	15,424.2	218.2	671.3	73.2	26.8	25.1	0.68
Gabon	Middle SDI	Mixed	1,764.9	290.8	2,251.8	44.5	55.5	16.7	0.53
Gambia	Low SDI	Mixed	2,149.4	257.6	3,355.6	34.0	66.0	21.6	0.80
Georgia	High-middle SDI	Prevention-intensive	5,917.8	297.9	737.0	70.8	29.2	11.6	0.75
Germany	High SDI	Mixed	14,735.2	190.9	711.2	68.8	31.2	25.1	0.79
Ghana	Low-middle SDI	Mortality-intensive	2,648.9	235.6	3,200.9	30.7	69.3	15.9	0.81
Greece	High-middle SDI	Mixed	6,645.1	280.2	765.7	75.6	24.4	22.8	0.69
Greenland	High SDI	Prevention-intensive	27,003.8	195.4	369.4	76.9	23.1	4.7	0.88
Grenada	Middle SDI	Long-term-care-intensive	4,030.4	241.2	996.1	62.5	37.5	16.6	0.80
Guam	High-middle SDI	Rehabilitation-intensive	1,632.0	430.1	998.0	73.0	27.0	9.6	0.80
Guatemala	Low-middle SDI	Rehabilitation-intensive	3,971.4	406.0	960.8	70.8	29.2	6.2	0.75
Guinea	Low SDI	Mixed	1,432.5	295.4	5,476.1	25.9	74.1	16.4	0.78
Guinea-Bissau	Low SDI	Mortality-intensive	1,454.7	286.7	4,631.3	27.0	73.0	11.0	0.74
Guyana	Middle SDI	Mortality-intensive	2,267.4	313.2	2,589.9	43.1	56.9	11.2	0.53
Haiti	Low SDI	Mortality-intensive	1,067.0	307.4	2,882.8	38.5	61.5	6.9	0.68
Honduras	Low-middle SDI	Rehabilitation-intensive	2,035.2	454.1	1,137.0	73.4	26.6	6.9	0.89
Hungary	High-middle SDI	Prevention-intensive	10,400.0	212.6	589.4	71.0	29.0	15.4	0.86

Table S1. Country-level raw indicators in 2023

Country	SDI quintile	Archetype	Incidence rate	YLDs per 1,000 falls	Deaths per 100,000 falls	YLD share of DALYs (%)	YLL share of DALYs (%)	85+ share of YLDs (%)	Female-to-male YLD intensity ratio
Iceland	High SDI	Mixed	12,011.1	240.9	655.1	75.4	24.6	19.4	0.61
India	Low-middle SDI	Mortality-intensive	5,496.9	269.1	1,944.0	45.2	54.8	9.4	0.66
Indonesia	Middle SDI	Mixed	2,532.2	313.4	1,413.1	53.9	46.1	8.0	0.58
Iran (Islamic Republic of)	Middle SDI	Mixed	2,081.6	331.1	734.3	74.9	25.1	8.4	0.62
Iraq	Middle SDI	Mixed	1,156.9	397.5	1,258.1	64.5	35.5	5.4	0.69
Ireland	High SDI	Prevention-intensive	8,485.3	285.3	535.0	80.4	19.6	15.9	0.64
Israel	High-middle SDI	Mixed	4,746.4	350.9	621.6	82.1	17.9	16.5	0.66
Italy	High-middle SDI	Mixed	8,530.0	223.7	847.1	69.8	30.2	24.6	0.64
Jamaica	Middle SDI	Long-term-care-intensive	1,686.3	349.3	1,329.3	66.3	33.7	17.8	0.69
Japan	High SDI	Mixed	5,405.5	250.9	795.0	73.1	26.9	27.5	1.09
Jordan	High-middle SDI	Mixed	1,279.3	378.7	1,236.8	64.9	35.1	8.8	0.79
Kazakhstan	High-middle SDI	Rehabilitation-intensive	2,108.1	400.5	691.3	74.9	25.1	5.0	0.84
Kenya	Low-middle SDI	Mortality-intensive	2,066.5	261.4	3,537.1	31.6	68.4	14.3	0.81
Kiribati	Low-middle SDI	Rehabilitation-intensive	417.7	564.0	1,136.3	74.4	25.6	8.1	0.79
Kuwait	High SDI	Rehabilitation-intensive	1,802.8	430.9	716.0	78.7	21.3	5.5	0.71
Kyrgyzstan	Low-middle SDI	Rehabilitation-intensive	1,072.9	443.6	726.5	75.3	24.7	4.3	0.85
Lao People's Democratic Republic	Low-middle SDI	Mortality-intensive	1,389.8	393.0	3,625.3	38.0	62.0	9.2	0.66
Latvia	High SDI	Mixed	6,185.1	348.9	616.2	77.7	22.3	12.6	0.91
Lebanon	High-middle SDI	Long-term-care-intensive	4,118.6	262.3	1,023.1	66.3	33.7	19.3	0.62
Lesotho	Low-middle SDI	Mixed	695.3	381.9	2,379.9	49.5	50.5	10.5	0.61
Liberia	Low SDI	Mortality-intensive	1,764.6	274.2	4,398.7	27.1	72.9	14.3	0.76
Libya	High-middle SDI	Rehabilitation-intensive	1,211.8	459.0	1,375.2	68.7	31.3	9.2	0.59
Lithuania	High SDI	Prevention-intensive	8,235.3	299.3	619.9	75.5	24.5	14.0	0.86
Luxembourg	High SDI	Mixed	13,834.0	215.3	650.4	73.2	26.8	21.2	0.66
Madagascar	Low SDI	Mortality-intensive	1,370.9	310.5	5,995.4	23.3	76.7	8.3	0.89
Malawi	Low SDI	Mortality-intensive	2,268.0	265.6	4,967.6	24.0	76.0	15.1	0.82
Malaysia	High-middle SDI	Mixed	1,879.6	331.3	1,751.0	53.2	46.8	9.3	0.70
Maldives	Middle SDI	Mixed	2,694.4	323.9	1,751.3	53.4	46.6	7.3	0.62

Table S1. Country-level raw indicators in 2023

Country	SDI quintile	Archetype	Incidence rate	YLDs per 1,000 falls	Deaths per 100,000 falls	YLD share of DALYs (%)	YLL share of DALYs (%)	85+ share of YLDs (%)	Female-to-male YLD intensity ratio
Mali	Low SDI	Mortality-intensive	1,454.1	272.1	4,381.4	27.0	73.0	12.6	0.81
Malta	High-middle SDI	Mixed	8,182.7	254.3	594.4	77.4	22.6	17.9	0.59
Marshall Islands	Low-middle SDI	Mortality-intensive	1,239.0	389.3	3,053.7	38.8	61.2	2.5	0.70
Mauritania	Low-middle SDI	Mortality-intensive	1,865.1	251.0	4,026.4	27.3	72.7	15.2	0.79
Mauritius	High-middle SDI	Rehabilitation-intensive	1,683.1	423.6	1,462.6	62.0	38.0	7.8	0.74
Mexico	Middle SDI	Rehabilitation-intensive	4,723.5	405.9	573.9	81.6	18.4	9.2	0.86
Micronesia (Federated States of)	Low-middle SDI	Mixed	3,017.3	295.2	1,520.8	50.8	49.2	5.3	0.78
Monaco	High SDI	Mixed	5,639.7	328.8	760.8	78.6	21.4	16.6	0.60
Mongolia	Low-middle SDI	Rehabilitation-intensive	1,568.9	413.7	997.3	66.7	33.3	5.4	0.63
Montenegro	High-middle SDI	Prevention-intensive	5,690.5	271.9	648.8	73.8	26.2	10.6	0.75
Morocco	Low-middle SDI	Mortality-intensive	820.3	454.8	3,455.3	45.0	55.0	6.9	0.86
Mozambique	Low SDI	Mixed	2,619.2	278.6	2,373.0	44.0	56.0	17.0	0.91
Myanmar	Low-middle SDI	Mortality-intensive	2,132.5	416.0	3,181.9	42.7	57.3	8.6	0.42
Namibia	Low-middle SDI	Mixed	717.9	401.7	1,605.2	60.9	39.1	11.4	0.62
Nauru	Middle SDI	Mixed	1,836.4	361.3	1,964.6	46.5	53.5	1.8	0.72
Nepal	Low SDI	Mixed	2,306.7	403.0	2,243.1	52.8	47.2	9.0	0.71
Netherlands	High SDI	Mixed	24,538.9	171.1	632.6	70.3	29.7	23.7	0.63
New Zealand	High SDI	Mixed	11,814.9	194.4	626.5	72.9	27.1	22.1	0.75
Nicaragua	Low-middle SDI	Rehabilitation-intensive	3,820.2	364.6	833.7	73.4	26.6	9.1	0.82
Niger	Low SDI	Mortality-intensive	1,698.5	277.4	5,171.7	23.2	76.8	8.7	0.83
Nigeria	Low-middle SDI	Mortality-intensive	2,172.9	247.3	2,248.6	40.0	60.0	15.8	0.76
Niue	High-middle SDI	Mixed	1,718.0	329.9	1,905.8	55.6	44.4	16.1	0.67
North Macedonia	High-middle SDI	Mixed	3,759.1	307.1	668.5	75.2	24.8	9.2	0.76
Northern Mariana Islands	High-middle SDI	Mixed	2,423.0	364.6	1,226.8	61.9	38.1	4.1	1.07
Norway	High SDI	Mixed	17,553.2	181.6	648.6	70.5	29.5	23.7	0.67
Oman	High-middle SDI	Mixed	3,090.0	279.0	725.0	69.9	30.1	6.1	0.67

Table S1. Country-level raw indicators in 2023

Country	SDI quintile	Archetype	Incidence rate	YLDs per 1,000 falls	Deaths per 100,000 falls	YLD share of DALYs (%)	YLL share of DALYs (%)	85+ share of YLDs (%)	Female-to-male YLD intensity ratio
Pakistan	Low-middle SDI	Mortality-intensive	1,152.9	384.1	3,707.0	39.0	61.0	9.2	0.67
Palau	High-middle SDI	Mixed	3,597.2	340.0	1,642.2	55.1	44.9	4.0	1.10
Palestine	Middle SDI	Mixed	1,798.4	333.8	1,301.7	61.2	38.8	9.5	0.72
Panama	Middle SDI	Rehabilitation-intensive	2,447.5	508.0	739.6	82.6	17.4	10.9	0.97
Papua New Guinea	Low SDI	Mortality-intensive	1,070.8	390.8	3,296.4	38.9	61.1	4.9	0.79
Paraguay	Middle SDI	Mixed	3,117.1	348.9	1,094.6	68.2	31.8	9.1	0.59
Peru	Middle SDI	Rehabilitation-intensive	3,136.9	396.9	1,005.3	72.4	27.6	10.0	0.57
Philippines	Middle SDI	Mixed	1,696.8	340.4	1,898.6	51.2	48.8	8.3	0.65
Poland	High-middle SDI	Prevention-intensive	7,138.7	263.0	772.6	70.1	29.9	14.8	0.68
Portugal	High-middle SDI	Mixed	7,198.5	248.4	656.3	75.2	24.8	22.0	0.65
Puerto Rico	High SDI	Long-term-care-intensive	4,613.4	299.2	900.7	71.5	28.5	16.8	0.70
Qatar	High SDI	Rehabilitation-intensive	1,459.1	563.7	923.1	77.6	22.4	3.2	1.16
Republic of Korea	High SDI	Prevention-intensive	8,651.4	230.8	395.7	80.0	20.0	14.0	0.84
Republic of Moldova	High-middle SDI	Rehabilitation-intensive	2,959.1	401.5	586.4	77.2	22.8	9.3	1.21
Romania	High-middle SDI	Rehabilitation-intensive	4,651.8	346.0	600.9	76.6	23.4	9.2	1.02
Russian Federation	High-middle SDI	Rehabilitation-intensive	3,916.2	380.1	552.7	79.5	20.5	8.1	0.79
Rwanda	Low SDI	Mortality-intensive	1,853.9	278.4	3,945.6	28.7	71.3	11.5	0.92
Saint Kitts and Nevis	High-middle SDI	Mixed	2,653.4	252.8	1,233.5	59.4	40.6	15.4	0.91
Saint Lucia	Middle SDI	Mixed	1,884.8	359.2	1,171.8	68.8	31.2	13.8	0.72
Saint Vincent and the Grenadines	Middle SDI	Mixed	2,015.4	382.2	1,739.9	60.2	39.8	13.8	0.91
Samoa	Low-middle SDI	Mixed	1,585.2	375.7	1,954.3	55.7	44.3	15.3	0.55
San Marino	High SDI	Mixed	6,325.1	334.9	475.6	86.1	13.9	19.3	0.70
Sao Tome and Principe	Low-middle SDI	Mortality-intensive	2,489.0	265.1	2,683.1	36.2	63.8	14.2	0.84
Saudi Arabia	High SDI	Rehabilitation-intensive	1,639.0	374.7	921.3	69.5	30.5	4.2	0.67
Senegal	Low SDI	Mixed	1,700.7	286.1	3,925.6	31.9	68.1	16.9	0.80
Serbia	High-middle SDI	Mixed	3,940.1	302.6	687.1	74.3	25.7	8.6	0.91
Seychelles	High-middle SDI	Rehabilitation-intensive	1,259.1	480.4	1,411.0	65.3	34.7	7.9	0.94
Sierra Leone	Low SDI	Mixed	2,260.1	255.8	4,797.3	25.4	74.6	16.6	0.79

Table S1. Country-level raw indicators in 2023

Country	SDI quintile	Archetype	Incidence rate	YLDs per 1,000 falls	Deaths per 100,000 falls	YLD share of DALYs (%)	YLL share of DALYs (%)	85+ share of YLDs (%)	Female-to-male YLD intensity ratio
Singapore	High SDI	Rehabilitation-intensive	4,131.1	333.5	455.8	83.0	17.0	11.5	1.06
Slovakia	High-middle SDI	Prevention-intensive	9,462.2	231.8	571.8	72.9	27.1	12.8	0.73
Slovenia	High SDI	Mixed	22,648.7	156.0	588.9	69.0	31.0	27.0	0.68
Solomon Islands	Low SDI	Mortality-intensive	1,246.9	419.3	3,714.8	37.2	62.8	4.6	0.70
Somalia	Low SDI	Mortality-intensive	1,571.4	290.3	2,513.5	42.6	57.4	12.1	0.84
South Africa	Middle SDI	Rehabilitation-intensive	578.9	508.8	1,542.3	65.5	34.5	8.9	0.97
South Sudan	Low SDI	Mortality-intensive	1,373.7	289.9	5,602.7	23.2	76.8	9.6	0.92
Spain	High-middle SDI	Mixed	7,163.9	326.3	544.9	82.6	17.4	20.7	0.77
Sri Lanka	Middle SDI	Mixed	3,592.3	290.8	1,285.2	57.4	42.6	11.5	0.62
Sudan	Low-middle SDI	Mixed	1,060.0	435.3	2,154.0	53.7	46.3	4.9	0.65
Suriname	Middle SDI	Mixed	2,609.7	265.6	1,337.5	58.9	41.1	15.7	0.64
Sweden	High SDI	Mixed	13,311.9	222.1	655.5	74.0	26.0	22.0	0.73
Switzerland	High SDI	Mixed	16,515.3	209.9	621.6	74.7	25.3	24.6	0.63
Syrian Arab Republic	Middle SDI	Rehabilitation-intensive	1,109.7	421.5	1,161.8	70.6	29.4	5.4	0.71
Taiwan	High SDI	Mixed	6,669.1	399.0	517.2	83.5	16.5	9.3	0.92
Tajikistan	Low-middle SDI	Rehabilitation-intensive	1,101.4	528.8	999.2	71.6	28.4	4.6	0.68
Thailand	Middle SDI	Rehabilitation-intensive	2,815.2	384.6	959.3	70.4	29.6	12.0	0.53
Timor-Leste	Low SDI	Mortality-intensive	1,921.5	340.4	2,419.6	44.8	55.2	7.1	0.62
Togo	Low SDI	Mortality-intensive	1,617.8	265.9	3,273.3	32.3	67.7	11.5	0.82
Tokelau	Middle SDI	Long-term-care-intensive	1,348.8	360.1	2,325.9	54.0	46.0	16.8	0.62
Tonga	Middle SDI	Mixed	1,382.8	354.2	1,900.4	56.0	44.0	15.2	0.50
Trinidad and Tobago	High-middle SDI	Mixed	2,043.5	304.9	1,375.7	60.0	40.0	9.0	0.62
Tunisia	Middle SDI	Long-term-care-intensive	4,900.0	209.5	1,273.7	51.2	48.8	13.5	0.69
Turkmenistan	Middle SDI	Rehabilitation-intensive	698.8	577.4	751.8	78.5	21.5	5.3	0.68
Tuvalu	Low-middle SDI	Mixed	1,733.2	423.3	2,163.7	52.4	47.6	5.0	0.81
Türkiye	High-middle SDI	Mixed	3,257.5	244.5	1,077.3	61.7	38.3	14.1	0.66

Table S1. Country-level raw indicators in 2023

Country	SDI quintile	Archetype	Incidence rate	YLDs per 1,000 falls	Deaths per 100,000 falls	YLD share of DALYs (%)	YLL share of DALYs (%)	85+ share of YLDs (%)	Female-to-male YLD intensity ratio
Uganda	Low SDI	Mortality-intensive	1,751.0	293.1	3,647.7	34.3	65.7	14.6	0.93
Ukraine	High-middle SDI	Rehabilitation-intensive	2,077.6	465.3	528.6	81.5	18.5	7.4	0.97
United Arab Emirates	High SDI	Rehabilitation-intensive	843.6	750.8	1,942.6	68.1	31.9	1.6	0.35
United Kingdom	High SDI	Mixed	9,128.7	237.4	805.8	70.3	29.7	20.6	0.70
United Republic of Tanzania	Low SDI	Mixed	2,258.6	272.7	2,915.0	38.0	62.0	17.6	0.88
United States Virgin Islands	High SDI	Mixed	2,345.5	337.1	1,634.1	61.2	38.8	14.8	0.62
United States of America	High SDI	Prevention-intensive	19,463.0	206.8	335.4	82.0	18.0	12.1	0.96
Uruguay	High-middle SDI	Long-term-care-intensive	3,975.7	258.8	1,110.0	65.6	34.4	22.4	0.59
Uzbekistan	Middle SDI	Rehabilitation-intensive	852.4	520.8	793.6	77.0	23.0	5.9	0.70
Vanuatu	Low-middle SDI	Mixed	1,133.0	419.1	2,061.7	55.2	44.8	9.5	0.75
Venezuela (Bolivarian Republic of)	Low-middle SDI	Rehabilitation-intensive	2,044.4	518.1	1,326.9	71.4	28.6	6.9	0.79
Viet Nam	Middle SDI	Mixed	6,339.2	236.1	1,304.9	53.6	46.4	20.7	0.59
Yemen	Low SDI	Mortality-intensive	909.3	409.5	2,496.3	48.4	51.6	4.9	0.77
Zambia	Low-middle SDI	Mortality-intensive	2,285.8	267.1	3,584.4	30.1	69.9	8.9	0.78
Zimbabwe	Low-middle SDI	Mixed	772.3	371.6	2,153.4	51.1	48.9	16.0	0.63

Country-level indicators are based on adults aged 60 years or older in 2023. YLDs per 1,000 falls, deaths per 100,000 falls, YLD/YLL shares, and female-to-male ratios are descriptive structural indicators derived from aggregated counts; no uncertainty intervals are shown.

Table S2. Country-level estimated annual percentage changes for main indicators, 1990-2023

Country	SDI quintile	Profile	Incidence rate EAPC (%)	YLDs per 1,000 falls EAPC (%)	Deaths per 100,000 falls EAPC (%)	85+ share of YLDs EAPC (%)
Afghanistan	Low SDI	Mortality-intensive	1.423	-0.900	-1.889	1.747
Albania	Middle SDI	Rehabilitation-intensive	1.457	-0.529	-1.862	0.344
Algeria	Middle SDI	Mixed	0.925	-0.832	-0.644	4.457
American Samoa	High-middle SDI	Mixed	1.006	-0.077	-0.172	1.951
Andorra	High SDI	Mixed	1.054	-0.155	0.089	2.778
Angola	Low-middle SDI	Mortality-intensive	1.396	-0.108	-1.331	2.138
Antigua and Barbuda	High-middle SDI	Mixed	0.762	-0.090	-1.528	-1.235
Argentina	High-middle SDI	Long-term-care-intensive	-0.420	-0.040	0.230	0.992
Armenia	Middle SDI	Rehabilitation-intensive	-1.008	-1.130	0.305	2.929
Australia	High SDI	Mixed	2.246	-0.615	1.512	1.965
Austria	High SDI	Mixed	0.756	-0.222	0.399	1.339
Azerbaijan	Middle SDI	Rehabilitation-intensive	0.774	-1.071	-1.998	-0.395
Bahamas	High-middle SDI	Mixed	0.864	0.022	-0.203	-0.298
Bahrain	High-middle SDI	Rehabilitation-intensive	-0.220	0.557	-2.192	1.454
Bangladesh	Low-middle SDI	Rehabilitation-intensive	2.665	-0.410	-2.204	-0.283
Barbados	High-middle SDI	Long-term-care-intensive	1.120	-0.211	-1.189	-0.353
Belarus	High-middle SDI	Mixed	2.330	-1.463	-0.809	1.474
Belgium	High SDI	Mixed	1.682	-0.249	0.202	1.487
Belize	Low-middle SDI	Mixed	1.315	-0.130	-0.860	-0.768
Benin	Low SDI	Mixed	0.464	-0.018	-1.332	0.791
Bermuda	High SDI	Rehabilitation-intensive	1.283	0.229	-0.833	0.965
Bhutan	Low-middle SDI	Mortality-intensive	3.468	-0.545	-2.449	2.918
Bolivia (Plurinational State of)	Low-middle SDI	Mixed	1.865	-0.674	-1.871	1.390
Bosnia and Herzegovina	High-middle SDI	Rehabilitation-intensive	0.695	-0.231	-0.566	0.966
Botswana	Middle SDI	Long-term-care-intensive	0.014	0.027	-0.819	3.423
Brazil	Middle SDI	Mixed	0.650	-0.958	1.555	2.303
Brunei Darussalam	High-middle SDI	Mixed	-0.166	-0.243	-1.381	-1.400
Bulgaria	High-middle SDI	Rehabilitation-intensive	0.202	-0.634	0.100	4.592

Table S2. Country-level estimated annual percentage changes for main indicators, 1990-2023

Country	SDI quintile	Profile	Incidence rate EAPC (%)	YLDs per 1,000 falls EAPC (%)	Deaths per 100,000 falls EAPC (%)	85+ share of YLDs EAPC (%)
Burkina Faso	Low SDI	Mixed	0.490	0.222	-0.606	1.804
Burundi	Low SDI	Mortality-intensive	0.292	0.434	-0.847	1.110
Cabo Verde	Low-middle SDI	Long-term-care-intensive	1.754	-0.765	-1.657	0.844
Cambodia	Low-middle SDI	Mixed	2.172	-0.401	-2.510	1.321
Cameroon	Low-middle SDI	Mortality-intensive	0.495	0.077	-0.961	0.397
Canada	High SDI	Mixed	2.166	-0.088	-0.022	0.937
Central African Republic	Low SDI	Mortality-intensive	-0.427	0.382	-0.410	1.379
Chad	Low SDI	Mortality-intensive	0.243	0.110	-0.086	-0.173
Chile	High-middle SDI	Long-term-care-intensive	1.556	-0.994	-0.492	1.636
China	High-middle SDI	Mixed	2.178	-0.764	-1.001	3.020
Colombia	Middle SDI	Rehabilitation-intensive	-0.230	0.040	-1.206	1.500
Comoros	Low-middle SDI	Long-term-care-intensive	1.659	-0.089	-1.670	3.045
Congo	Low-middle SDI	Mortality-intensive	0.443	0.202	-0.667	3.027
Cook Islands	High-middle SDI	Rehabilitation-intensive	1.152	0.791	-0.805	1.161
Costa Rica	Middle SDI	Prevention-intensive	0.193	0.079	-1.143	0.193
Croatia	High-middle SDI	Mixed	2.368	-0.730	-1.134	2.261
Cuba	Middle SDI	Mixed	2.417	-0.515	-1.280	1.133
Cyprus	High SDI	Prevention-intensive	0.736	0.108	-1.793	5.153
Czechia	High SDI	Prevention-intensive	-2.198	0.864	-0.948	0.144
Côte d'Ivoire	Low SDI	Mortality-intensive	0.329	0.203	-0.519	1.973
Democratic People's Republic of Korea	Low-middle SDI	Rehabilitation-intensive	0.332	0.644	1.052	1.421
Democratic Republic of the Congo	Low SDI	Mortality-intensive	0.385	0.249	0.174	3.367
Denmark	High SDI	Mixed	-1.929	0.926	-0.830	-0.089
Djibouti	Low-middle SDI	Mortality-intensive	1.125	-0.148	-1.437	1.166
Dominica	High-middle SDI	Long-term-care-intensive	1.656	-0.144	-1.808	0.622
Dominican Republic	Middle SDI	Mixed	2.157	-0.678	-1.482	1.003

Table S2. Country-level estimated annual percentage changes for main indicators, 1990-2023

Country	SDI quintile	Profile	Incidence rate EAPC (%)	YLDs per 1,000 falls EAPC (%)	Deaths per 100,000 falls EAPC (%)	85+ share of YLDs EAPC (%)
Ecuador	Middle SDI	Rehabilitation-intensive	1.726	-0.893	-1.444	0.941
Egypt	Low-middle SDI	Mixed	1.073	-0.535	-1.529	-0.404
El Salvador	Low-middle SDI	Rehabilitation-intensive	1.572	-0.644	-1.617	0.939
Equatorial Guinea	Middle SDI	Mortality-intensive	2.116	-0.509	-1.930	3.148
Eritrea	Low SDI	Mortality-intensive	1.795	0.098	-0.810	2.910
Estonia	High SDI	Prevention-intensive	0.081	-0.711	-0.558	2.090
Eswatini	Low-middle SDI	Mixed	-0.727	0.212	-0.987	-0.737
Ethiopia	Low SDI	Mortality-intensive	0.608	-0.058	-0.422	5.290
Fiji	Middle SDI	Mixed	0.767	-0.182	-1.012	0.159
Finland	High SDI	Mixed	0.343	-0.108	-0.296	1.273
France	High SDI	Mixed	0.575	0.119	-0.449	1.052
Gabon	Middle SDI	Mixed	-0.114	0.011	-1.182	0.910
Gambia	Low SDI	Mixed	1.158	-0.116	-0.904	1.883
Georgia	High-middle SDI	Prevention-intensive	3.480	-1.799	0.836	2.453
Germany	High SDI	Mixed	1.413	-0.560	0.382	1.339
Ghana	Low-middle SDI	Mortality-intensive	0.829	-0.028	-0.361	1.339
Greece	High-middle SDI	Mixed	-0.269	0.060	3.522	2.981
Greenland	High SDI	Prevention-intensive	0.713	-0.131	-1.074	0.466
Grenada	Middle SDI	Long-term-care-intensive	1.179	-0.303	-2.239	-2.483
Guam	High-middle SDI	Rehabilitation-intensive	0.875	0.382	-1.482	3.858
Guatemala	Low-middle SDI	Rehabilitation-intensive	0.892	-0.103	-2.027	2.493
Guinea	Low SDI	Mixed	0.289	0.150	-0.001	1.988
Guinea-Bissau	Low SDI	Mortality-intensive	0.577	0.053	-0.795	1.050
Guyana	Middle SDI	Mortality-intensive	-0.082	0.341	-0.164	0.044
Haiti	Low SDI	Mortality-intensive	0.653	-0.089	-0.468	0.562
Honduras	Low-middle SDI	Rehabilitation-intensive	1.300	-1.029	-1.225	-0.494
Hungary	High-middle SDI	Prevention-intensive	-2.052	0.339	-1.560	1.021
Iceland	High SDI	Mixed	0.473	0.142	1.124	1.104

Table S2. Country-level estimated annual percentage changes for main indicators, 1990-2023

Country	SDI quintile	Profile	Incidence rate EAPC (%)	YLDs per 1,000 falls EAPC (%)	Deaths per 100,000 falls EAPC (%)	85+ share of YLDs EAPC (%)
India	Low-middle SDI	Mortality-intensive	0.255	0.117	-0.178	1.407
Indonesia	Middle SDI	Mixed	-0.219	-0.268	-0.627	-0.070
Iran (Islamic Republic of)	Middle SDI	Mixed	0.935	-0.843	-0.492	4.128
Iraq	Middle SDI	Mixed	0.368	-0.828	-1.514	-1.544
Ireland	High SDI	Prevention-intensive	0.044	0.639	-0.692	0.939
Israel	High-middle SDI	Mixed	0.241	0.475	-0.224	1.504
Italy	High-middle SDI	Mixed	-0.944	0.554	1.228	1.294
Jamaica	Middle SDI	Long-term-care-intensive	1.817	-0.542	-0.392	1.213
Japan	High SDI	Mixed	0.409	-0.240	1.746	3.284
Jordan	High-middle SDI	Mixed	0.644	-0.412	-2.200	0.310
Kazakhstan	High-middle SDI	Rehabilitation-intensive	2.352	-1.618	-1.887	-0.127
Kenya	Low-middle SDI	Mortality-intensive	0.414	-0.043	-0.880	0.365
Kiribati	Low-middle SDI	Rehabilitation-intensive	-0.034	0.323	-0.772	0.663
Kuwait	High SDI	Rehabilitation-intensive	-0.815	-0.939	-1.118	1.428
Kyrgyzstan	Low-middle SDI	Rehabilitation-intensive	-0.420	-1.139	-0.987	0.731
Lao People's Democratic Republic	Low-middle SDI	Mortality-intensive	1.512	-0.224	-1.381	3.114
Latvia	High SDI	Mixed	-1.068	-0.394	-0.139	1.336
Lebanon	High-middle SDI	Long-term-care-intensive	2.972	-1.143	-2.420	2.503
Lesotho	Low-middle SDI	Mixed	-0.287	-0.102	-0.612	-1.600
Liberia	Low SDI	Mortality-intensive	0.460	0.060	-0.796	1.854
Libya	High-middle SDI	Rehabilitation-intensive	0.506	-0.458	-0.227	-0.102
Lithuania	High SDI	Prevention-intensive	1.181	-1.283	0.034	2.391
Luxembourg	High SDI	Mixed	1.076	-0.298	0.186	1.851
Madagascar	Low SDI	Mortality-intensive	0.234	-0.024	-0.934	-0.302
Malawi	Low SDI	Mortality-intensive	1.109	0.039	-0.807	2.152
Malaysia	High-middle SDI	Mixed	0.372	-0.069	-1.378	-1.614
Maldives	Middle SDI	Mixed	1.989	-0.629	-1.974	4.308

Table S2. Country-level estimated annual percentage changes for main indicators, 1990-2023

Country	SDI quintile	Profile	Incidence rate EAPC (%)	YLDs per 1,000 falls EAPC (%)	Deaths per 100,000 falls EAPC (%)	85+ share of YLDs EAPC (%)
Mali	Low SDI	Mortality-intensive	0.342	0.079	-0.474	1.014
Malta	High-middle SDI	Mixed	-0.153	0.445	0.034	1.237
Marshall Islands	Low-middle SDI	Mortality-intensive	1.044	0.294	-1.019	-2.408
Mauritania	Low-middle SDI	Mortality-intensive	0.416	-0.037	-0.607	1.592
Mauritius	High-middle SDI	Rehabilitation-intensive	2.160	-0.138	-0.637	1.907
Mexico	Middle SDI	Rehabilitation-intensive	-0.552	0.338	-1.488	0.857
Micronesia (Federated States of)	Low-middle SDI	Mixed	1.521	-0.240	-1.945	-0.079
Monaco	High SDI	Mixed	0.059	0.319	0.400	0.215
Mongolia	Low-middle SDI	Rehabilitation-intensive	1.104	-0.303	-2.049	-0.229
Montenegro	High-middle SDI	Prevention-intensive	-0.179	-0.397	-0.280	-0.589
Morocco	Low-middle SDI	Mortality-intensive	0.367	-0.522	-0.449	-0.275
Mozambique	Low SDI	Mixed	1.435	-0.254	-1.218	1.119
Myanmar	Low-middle SDI	Mortality-intensive	1.603	-0.051	-1.695	2.859
Namibia	Low-middle SDI	Mixed	0.464	0.061	-1.016	2.545
Nauru	Middle SDI	Mixed	-0.452	-0.043	-0.837	1.105
Nepal	Low SDI	Mixed	0.686	-0.143	-0.546	1.194
Netherlands	High SDI	Mixed	3.433	-0.430	-0.392	0.702
New Zealand	High SDI	Mixed	0.623	-0.129	1.538	1.172
Nicaragua	Low-middle SDI	Rehabilitation-intensive	1.788	-0.758	-2.110	0.228
Niger	Low SDI	Mortality-intensive	0.746	0.022	-0.441	0.784
Nigeria	Low-middle SDI	Mortality-intensive	0.845	-0.257	-1.381	1.169
Niue	High-middle SDI	Mixed	0.450	-0.113	-1.596	-0.800
North Macedonia	High-middle SDI	Mixed	3.108	-1.609	-3.013	0.422
Northern Mariana Islands	High-middle SDI	Mixed	0.727	0.079	-0.779	0.931
Norway	High SDI	Mixed	0.290	-0.022	0.091	0.941
Oman	High-middle SDI	Mixed	0.474	-0.564	-1.743	-0.549

Table S2. Country-level estimated annual percentage changes for main indicators, 1990-2023

Country	SDI quintile	Profile	Incidence rate EAPC (%)	YLDs per 1,000 falls EAPC (%)	Deaths per 100,000 falls EAPC (%)	85+ share of YLDs EAPC (%)
Pakistan	Low-middle SDI	Mortality-intensive	-0.083	-0.033	-1.344	0.290
Palau	High-middle SDI	Mixed	0.085	0.450	-1.032	-0.505
Palestine	Middle SDI	Mixed	0.737	-0.330	-2.301	0.630
Panama	Middle SDI	Rehabilitation-intensive	-0.646	0.655	-1.441	-0.134
Papua New Guinea	Low SDI	Mortality-intensive	0.059	0.435	0.516	1.678
Paraguay	Middle SDI	Mixed	1.705	-1.423	-0.107	0.669
Peru	Middle SDI	Rehabilitation-intensive	1.921	-0.405	-1.700	1.020
Philippines	Middle SDI	Mixed	0.252	-0.225	0.351	0.826
Poland	High-middle SDI	Prevention-intensive	-0.138	0.026	-0.760	1.070
Portugal	High-middle SDI	Mixed	1.532	-1.188	0.563	3.693
Puerto Rico	High SDI	Long-term-care-intensive	1.947	-0.419	-1.446	1.281
Qatar	High SDI	Rehabilitation-intensive	0.549	-0.092	-3.902	0.397
Republic of Korea	High SDI	Prevention-intensive	1.952	-1.528	-1.553	3.977
Republic of Moldova	High-middle SDI	Rehabilitation-intensive	-0.416	-0.482	-0.200	3.128
Romania	High-middle SDI	Rehabilitation-intensive	0.983	-1.420	-0.694	3.436
Russian Federation	High-middle SDI	Rehabilitation-intensive	1.198	-1.032	-0.251	2.403
Rwanda	Low SDI	Mortality-intensive	1.287	0.110	-1.867	2.335
Saint Kitts and Nevis	High-middle SDI	Mixed	1.469	-0.069	-1.279	1.125
Saint Lucia	Middle SDI	Mixed	1.466	0.182	-0.979	2.327
Saint Vincent and the Grenadines	Middle SDI	Mixed	1.222	0.171	-0.578	0.710
Samoa	Low-middle SDI	Mixed	1.217	-0.008	-0.920	1.591
San Marino	High SDI	Mixed	0.382	0.083	-1.099	1.295
Sao Tome and Principe	Low-middle SDI	Mortality-intensive	1.400	-0.430	-1.317	0.524
Saudi Arabia	High SDI	Rehabilitation-intensive	-0.123	-0.044	-1.266	-0.887
Senegal	Low SDI	Mixed	0.579	0.125	-0.701	1.385
Serbia	High-middle SDI	Mixed	1.059	-0.649	-1.187	3.609
Seychelles	High-middle SDI	Rehabilitation-intensive	0.114	0.171	-1.862	-0.419

Table S2. Country-level estimated annual percentage changes for main indicators, 1990-2023

Country	SDI quintile	Profile	Incidence rate EAPC (%)	YLDs per 1,000 falls EAPC (%)	Deaths per 100,000 falls EAPC (%)	85+ share of YLDs EAPC (%)
Sierra Leone	Low SDI	Mixed	1.144	0.040	-0.716	0.579
Singapore	High SDI	Rehabilitation-intensive	0.434	-0.150	-1.441	1.708
Slovakia	High-middle SDI	Prevention-intensive	0.215	-0.377	-1.116	0.676
Slovenia	High SDI	Mixed	1.126	-0.436	-0.479	2.029
Solomon Islands	Low SDI	Mortality-intensive	-0.043	0.455	0.904	1.833
Somalia	Low SDI	Mortality-intensive	0.250	0.066	-1.624	-0.622
South Africa	Middle SDI	Rehabilitation-intensive	-0.621	-0.194	-0.805	-0.499
South Sudan	Low SDI	Mortality-intensive	0.012	-0.032	-0.561	1.061
Spain	High-middle SDI	Mixed	1.955	-0.385	0.509	3.027
Sri Lanka	Middle SDI	Mixed	1.664	-0.645	-3.329	0.975
Sudan	Low-middle SDI	Mixed	0.232	-0.438	-1.461	1.470
Suriname	Middle SDI	Mixed	1.669	-0.393	-1.626	-0.280
Sweden	High SDI	Mixed	0.510	-0.061	0.475	0.911
Switzerland	High SDI	Mixed	0.003	-0.172	0.248	1.094
Syrian Arab Republic	Middle SDI	Rehabilitation-intensive	0.801	-0.584	-1.950	-0.759
Taiwan	High SDI	Mixed	0.193	-0.248	1.479	4.141
Tajikistan	Low-middle SDI	Rehabilitation-intensive	0.364	-1.294	-1.767	-0.339
Thailand	Middle SDI	Rehabilitation-intensive	0.862	0.448	-2.174	0.752
Timor-Leste	Low SDI	Mortality-intensive	2.305	-0.491	-1.309	0.371
Togo	Low SDI	Mortality-intensive	0.068	-0.016	-0.851	0.076
Tokelau	Middle SDI	Long-term-care-intensive	1.612	-0.295	-1.504	2.026
Tonga	Middle SDI	Mixed	1.225	-0.255	-0.449	1.009
Trinidad and Tobago	High-middle SDI	Mixed	-0.073	0.258	-0.978	-0.369
Tunisia	Middle SDI	Long-term-care-intensive	2.271	-1.234	-1.464	2.857
Turkmenistan	Middle SDI	Rehabilitation-intensive	0.145	-0.612	-0.648	1.393
Tuvalu	Low-middle SDI	Mixed	1.679	0.160	-0.867	2.327
Türkiye	High-middle SDI	Mixed	4.335	-2.303	-4.045	1.713
Uganda	Low SDI	Mortality-intensive	1.025	0.145	-1.407	1.391

Table S2. Country-level estimated annual percentage changes for main indicators, 1990-2023

Country	SDI quintile	Profile	Incidence rate EAPC (%)	YLDs per 1,000 falls EAPC (%)	Deaths per 100,000 falls EAPC (%)	85+ share of YLDs EAPC (%)
Ukraine	High-middle SDI	Rehabilitation-intensive	-1.040	-0.379	-0.308	1.972
United Arab Emirates	High SDI	Rehabilitation-intensive	-1.752	1.417	-1.400	-6.524
United Kingdom	High SDI	Mixed	1.360	-0.417	1.019	1.305
United Republic of Tanzania	Low SDI	Mixed	1.245	0.021	-1.393	2.461
United States Virgin Islands	High SDI	Mixed	1.216	-0.296	-2.502	0.744
United States of America	High SDI	Prevention-intensive	2.161	-0.267	0.695	-0.088
Uruguay	High-middle SDI	Long-term-care-intensive	0.786	-0.517	0.551	1.636
Uzbekistan	Middle SDI	Rehabilitation-intensive	0.638	-1.417	-1.462	-0.683
Vanuatu	Low-middle SDI	Mixed	1.012	0.018	-1.026	1.871
Venezuela (Bolivarian Republic of)	Low-middle SDI	Rehabilitation-intensive	-0.783	0.277	-0.754	-0.216
Viet Nam	Middle SDI	Mixed	2.345	-0.406	-2.423	0.883
Yemen	Low SDI	Mortality-intensive	0.571	-0.342	-1.002	1.192
Zambia	Low-middle SDI	Mortality-intensive	1.336	0.045	-1.731	0.715
Zimbabwe	Low-middle SDI	Mixed	-1.263	0.481	0.149	0.003

EAPC was estimated using a log-linear regression model across annual country-specific values from 1990 to 2023. Positive values indicate an increasing annual trend; negative values indicate a decreasing annual trend.

Table S3. Country nursing priority profiles and sensitivity classifications

Country	SDI quintile	Primary profile	Age-standardized incidence	80+ sensitivity	P80 sensitivity	Number/Rate-only	Female model	Male model
Afghanistan	Low SDI	Mortality-intensive	Mortality-intensive	Mortality-intensive	Mixed	Mortality-intensive	Mixed	Mortality-intensive
Albania	Middle SDI	Rehabilitation-intensive	Rehabilitation-intensive	Rehabilitation-intensive	Rehabilitation-intensive	Rehabilitation-intensive	Rehabilitation-intensive	Rehabilitation-intensive
Algeria	Middle SDI	Mixed	Mixed	Mixed	Mixed	Rehabilitation-intensive	Mixed	Mixed
American Samoa	High-middle SDI	Mixed	Mixed	Mixed	Mixed	Mixed	Mixed	Mixed
Andorra	High SDI	Mixed	Mixed	Mixed	Mixed	Mixed	Mixed	Mixed
Angola	Low-middle SDI	Mortality-intensive	Mortality-intensive	Mortality-intensive	Mixed	Mixed	Mortality-intensive	Mortality-intensive
Antigua and Barbuda	High-middle SDI	Mixed	Mixed	Mixed	Mixed	Mixed	Mixed	Mixed
Argentina	High-middle SDI	Long-term-care-intensive	Long-term-care-intensive	Long-term-care-intensive	Mixed	Mixed	Long-term-care-intensive	Mixed
Armenia	Middle SDI	Rehabilitation-intensive	Rehabilitation-intensive	Rehabilitation-intensive	Rehabilitation-intensive	Mixed	Rehabilitation-intensive	Rehabilitation-intensive
Australia	High SDI	Mixed	Mixed	Mixed	Mixed	Mixed	Mixed	Mixed
Austria	High SDI	Mixed	Mixed	Mixed	Mixed	Mixed	Mixed	Mixed
Azerbaijan	Middle SDI	Rehabilitation-intensive	Rehabilitation-intensive	Rehabilitation-intensive	Rehabilitation-intensive	Rehabilitation-intensive	Rehabilitation-intensive	Rehabilitation-intensive
Bahamas	High-middle SDI	Mixed	Mixed	Mixed	Mixed	Mixed	Mixed	Mixed
Bahrain	High-middle SDI	Rehabilitation-intensive	Rehabilitation-intensive	Rehabilitation-intensive	Rehabilitation-intensive	Rehabilitation-intensive	Rehabilitation-intensive	Rehabilitation-intensive
Bangladesh	Low-middle SDI	Rehabilitation-intensive	Rehabilitation-intensive	Rehabilitation-intensive	Rehabilitation-intensive	Mixed	Rehabilitation-intensive	Mixed
Barbados	High-middle SDI	Long-term-care-intensive	Long-term-care-intensive	Long-term-care-intensive	Long-term-care-intensive	Mixed	Mixed	Mixed
Belarus	High-middle SDI	Mixed	Mixed	Mixed	Rehabilitation-intensive	Rehabilitation-intensive	Rehabilitation-intensive	Prevention-intensive
Belgium	High SDI	Mixed	Mixed	Mixed	Mixed	Mixed	Mixed	Mixed
Belize	Low-middle SDI	Mixed	Mixed	Mixed	Mixed	Mixed	Mixed	Mixed
Benin	Low SDI	Mixed	Mixed	Mixed	Mixed	Mortality-intensive	Mixed	Mortality-intensive
Bermuda	High SDI	Rehabilitation-intensive	Rehabilitation-intensive	Rehabilitation-intensive	Mixed	Mixed	Rehabilitation-intensive	Long-term-care-intensive
Bhutan	Low-middle SDI	Mortality-intensive	Mortality-intensive	Mixed	Mixed	Mortality-intensive	Mixed	Mixed
Bolivia (Plurinational State of)	Low-middle SDI	Mixed	Mixed	Mixed	Mixed	Mixed	Mixed	Mixed
Bosnia and Herzegovina	High-middle SDI	Rehabilitation-intensive	Rehabilitation-intensive	Rehabilitation-intensive	Mixed	Mixed	Rehabilitation-intensive	Mixed
Botswana	Middle SDI	Long-term-care-intensive	Long-term-care-intensive	Long-term-care-intensive	Long-term-care-intensive	Mixed	Long-term-care-intensive	Long-term-care-intensive

Table S3. Country nursing priority profiles and sensitivity classifications

Country	SDI quintile	Primary profile	Age-standardized incidence	80+ sensitivity	P80 sensitivity	Number/Rate-only	Female model	Male model
Brazil	Middle SDI	Mixed	Mixed	Mixed	Mixed	Mixed	Mixed	Mixed
Brunei Darussalam	High-middle SDI	Mixed	Mixed	Mixed	Mixed	Mixed	Mixed	Mixed
Bulgaria	High-middle SDI	Rehabilitation-intensive	Rehabilitation-intensive	Rehabilitation-intensive	Rehabilitation-intensive	Rehabilitation-intensive	Rehabilitation-intensive	Rehabilitation-intensive
Burkina Faso	Low SDI	Mixed	Mixed	Mixed	Mixed	Mortality-intensive	Mixed	Mixed
Burundi	Low SDI	Mortality-intensive	Mortality-intensive	Mortality-intensive	Mortality-intensive	Mortality-intensive	Mortality-intensive	Mortality-intensive
Cabo Verde	Low-middle SDI	Long-term-care-intensive	Long-term-care-intensive	Long-term-care-intensive	Long-term-care-intensive	Mixed	Mixed	Long-term-care-intensive
Cambodia	Low-middle SDI	Mixed	Mixed	Mixed	Mixed	Mixed	Mixed	Mixed
Cameroon	Low-middle SDI	Mortality-intensive	Mortality-intensive	Mortality-intensive	Mortality-intensive	Mortality-intensive	Mortality-intensive	Mortality-intensive
Canada	High SDI	Mixed	Mixed	Mixed	Mixed	Mixed	Mixed	Mixed
Central African Republic	Low SDI	Mortality-intensive	Mortality-intensive	Mortality-intensive	Mortality-intensive	Mortality-intensive	Mortality-intensive	Mortality-intensive
Chad	Low SDI	Mortality-intensive	Mortality-intensive	Mortality-intensive	Mortality-intensive	Mortality-intensive	Mortality-intensive	Mortality-intensive
Chile	High-middle SDI	Long-term-care-intensive	Mixed	Long-term-care-intensive	Mixed	Mixed	Long-term-care-intensive	Mixed
China	High-middle SDI	Mixed	Mixed	Mixed	Rehabilitation-intensive	Mixed	Mixed	Mixed
Colombia	Middle SDI	Rehabilitation-intensive	Rehabilitation-intensive	Rehabilitation-intensive	Rehabilitation-intensive	Rehabilitation-intensive	Rehabilitation-intensive	Mixed
Comoros	Low-middle SDI	Long-term-care-intensive	Long-term-care-intensive	Long-term-care-intensive	Long-term-care-intensive	Mixed	Long-term-care-intensive	Long-term-care-intensive
Congo	Low-middle SDI	Mortality-intensive	Mortality-intensive	Mortality-intensive	Mortality-intensive	Mortality-intensive	Mortality-intensive	Mortality-intensive
Cook Islands	High-middle SDI	Rehabilitation-intensive	Rehabilitation-intensive	Rehabilitation-intensive	Rehabilitation-intensive	Mixed	Rehabilitation-intensive	Rehabilitation-intensive
Costa Rica	Middle SDI	Prevention-intensive	Prevention-intensive	Prevention-intensive	Prevention-intensive	Mixed	Prevention-intensive	Prevention-intensive
Croatia	High-middle SDI	Mixed	Mixed	Mixed	Mixed	Mixed	Mixed	Mixed
Cuba	Middle SDI	Mixed	Mixed	Mixed	Mixed	Mixed	Mixed	Mixed
Cyprus	High SDI	Prevention-intensive	Prevention-intensive	Prevention-intensive	Prevention-intensive	Mixed	Prevention-intensive	Mixed
Czechia	High SDI	Prevention-intensive	Prevention-intensive	Mixed	Prevention-intensive	Mixed	Prevention-intensive	Prevention-intensive
Côte d'Ivoire	Low SDI	Mortality-intensive	Mortality-intensive	Mortality-intensive	Mortality-intensive	Mortality-intensive	Mortality-intensive	Mortality-intensive
Democratic People's Republic of Korea	Low-middle SDI	Rehabilitation-intensive	Mixed	Rehabilitation-intensive	Rehabilitation-intensive	Rehabilitation-intensive	Rehabilitation-intensive	Rehabilitation-intensive
Democratic Republic of the Congo	Low SDI	Mortality-intensive	Mortality-intensive	Mortality-intensive	Mortality-intensive	Mortality-intensive	Mortality-intensive	Mortality-intensive
Denmark	High SDI	Mixed	Mixed	Mixed	Mixed	Mixed	Mixed	Mixed

Table S3. Country nursing priority profiles and sensitivity classifications

Country	SDI quintile	Primary profile	Age-standardized incidence	80+ sensitivity	P80 sensitivity	Number/Rate-only	Female model	Male model
Djibouti	Low-middle SDI	Mortality-intensive	Mortality-intensive	Mortality-intensive	Mixed	Mixed	Mortality-intensive	Mixed
Dominica	High-middle SDI	Long-term-care-intensive	Long-term-care-intensive	Long-term-care-intensive	Long-term-care-intensive	Mixed	Long-term-care-intensive	Long-term-care-intensive
Dominican Republic	Middle SDI	Mixed	Mixed	Mixed	Mixed	Mixed	Mixed	Mixed
Ecuador	Middle SDI	Rehabilitation-intensive	Rehabilitation-intensive	Rehabilitation-intensive	Mixed	Mixed	Mixed	Rehabilitation-intensive
Egypt	Low-middle SDI	Mixed	Mixed	Mixed	Mixed	Rehabilitation-intensive	Mixed	Rehabilitation-intensive
El Salvador	Low-middle SDI	Rehabilitation-intensive	Rehabilitation-intensive	Rehabilitation-intensive	Mixed	Rehabilitation-intensive	Rehabilitation-intensive	Mixed
Equatorial Guinea	Middle SDI	Mortality-intensive	Mortality-intensive	Mortality-intensive	Mortality-intensive	Mortality-intensive	Mortality-intensive	Mortality-intensive
Eritrea	Low SDI	Mortality-intensive	Mortality-intensive	Mortality-intensive	Mortality-intensive	Mortality-intensive	Mortality-intensive	Mortality-intensive
Estonia	High SDI	Prevention-intensive	Prevention-intensive	Mixed	Prevention-intensive	Mixed	Mixed	Prevention-intensive
Eswatini	Low-middle SDI	Mixed	Mixed	Mixed	Mixed	Rehabilitation-intensive	Mixed	Rehabilitation-intensive
Ethiopia	Low SDI	Mortality-intensive	Mortality-intensive	Mixed	Mortality-intensive	Mortality-intensive	Mortality-intensive	Mixed
Fiji	Middle SDI	Mixed	Mixed	Mixed	Mixed	Mixed	Mixed	Mixed
Finland	High SDI	Mixed	Mixed	Mixed	Mixed	Mixed	Mixed	Mixed
France	High SDI	Mixed	Mixed	Mixed	Mixed	Mixed	Mixed	Mixed
Gabon	Middle SDI	Mixed	Mixed	Mortality-intensive	Mixed	Mixed	Mixed	Mixed
Gambia	Low SDI	Mixed	Mixed	Mixed	Mixed	Mortality-intensive	Mixed	Mixed
Georgia	High-middle SDI	Prevention-intensive	Prevention-intensive	Prevention-intensive	Mixed	Mixed	Rehabilitation-intensive	Prevention-intensive
Germany	High SDI	Mixed	Mixed	Mixed	Mixed	Mixed	Mixed	Mixed
Ghana	Low-middle SDI	Mortality-intensive	Mortality-intensive	Mixed	Mortality-intensive	Mortality-intensive	Mortality-intensive	Mortality-intensive
Greece	High-middle SDI	Mixed	Mixed	Mixed	Mixed	Mixed	Mixed	Mixed
Greenland	High SDI	Prevention-intensive	Prevention-intensive	Prevention-intensive	Prevention-intensive	Mixed	Prevention-intensive	Prevention-intensive
Grenada	Middle SDI	Long-term-care-intensive	Long-term-care-intensive	Long-term-care-intensive	Mixed	Mixed	Long-term-care-intensive	Mixed
Guam	High-middle SDI	Rehabilitation-intensive	Rehabilitation-intensive	Rehabilitation-intensive	Rehabilitation-intensive	Rehabilitation-intensive	Rehabilitation-intensive	Rehabilitation-intensive
Guatemala	Low-middle SDI	Rehabilitation-intensive	Mixed	Rehabilitation-intensive	Rehabilitation-intensive	Rehabilitation-intensive	Rehabilitation-intensive	Mixed
Guinea	Low SDI	Mixed	Mixed	Mortality-intensive	Mortality-intensive	Mortality-intensive	Mixed	Mixed
Guinea-Bissau	Low SDI	Mortality-intensive	Mortality-intensive	Mortality-intensive	Mortality-intensive	Mortality-intensive	Mortality-intensive	Mortality-intensive

Table S3. Country nursing priority profiles and sensitivity classifications

Country	SDI quintile	Primary profile	Age-standardized incidence	80+ sensitivity	P80 sensitivity	Number/Rate-only	Female model	Male model
Guyana	Middle SDI	Mortality-intensive	Mortality-intensive	Mortality-intensive	Mortality-intensive	Mortality-intensive	Mortality-intensive	Mortality-intensive
Haiti	Low SDI	Mortality-intensive	Mortality-intensive	Mortality-intensive	Mortality-intensive	Mortality-intensive	Mortality-intensive	Mortality-intensive
Honduras	Low-middle SDI	Rehabilitation-intensive	Rehabilitation-intensive	Rehabilitation-intensive	Rehabilitation-intensive	Rehabilitation-intensive	Rehabilitation-intensive	Rehabilitation-intensive
Hungary	High-middle SDI	Prevention-intensive	Prevention-intensive	Mixed	Prevention-intensive	Mixed	Prevention-intensive	Prevention-intensive
Iceland	High SDI	Mixed	Mixed	Mixed	Mixed	Mixed	Mixed	Mixed
India	Low-middle SDI	Mortality-intensive	Mixed	Mortality-intensive	Mixed	Mixed	Mortality-intensive	Mortality-intensive
Indonesia	Middle SDI	Mixed	Mixed	Mixed	Mixed	Mixed	Mixed	Mixed
Iran (Islamic Republic of)	Middle SDI	Mixed	Mixed	Mixed	Mixed	Mixed	Mixed	Rehabilitation-intensive
Iraq	Middle SDI	Mixed	Mixed	Mixed	Mixed	Rehabilitation-intensive	Rehabilitation-intensive	Rehabilitation-intensive
Ireland	High SDI	Prevention-intensive	Prevention-intensive	Prevention-intensive	Prevention-intensive	Mixed	Prevention-intensive	Mixed
Israel	High-middle SDI	Mixed	Mixed	Mixed	Rehabilitation-intensive	Mixed	Mixed	Mixed
Italy	High-middle SDI	Mixed	Mixed	Mixed	Mixed	Mixed	Mixed	Mixed
Jamaica	Middle SDI	Long-term-care-intensive	Long-term-care-intensive	Mixed	Long-term-care-intensive	Mixed	Long-term-care-intensive	Long-term-care-intensive
Japan	High SDI	Mixed	Long-term-care-intensive	Mixed	Long-term-care-intensive	Prevention-intensive	Mixed	Mixed
Jordan	High-middle SDI	Mixed	Mixed	Mixed	Mixed	Mixed	Rehabilitation-intensive	Mixed
Kazakhstan	High-middle SDI	Rehabilitation-intensive	Rehabilitation-intensive	Rehabilitation-intensive	Rehabilitation-intensive	Rehabilitation-intensive	Rehabilitation-intensive	Rehabilitation-intensive
Kenya	Low-middle SDI	Mortality-intensive	Mortality-intensive	Mortality-intensive	Mortality-intensive	Mortality-intensive	Mortality-intensive	Mortality-intensive
Kiribati	Low-middle SDI	Rehabilitation-intensive	Rehabilitation-intensive	Rehabilitation-intensive	Rehabilitation-intensive	Rehabilitation-intensive	Rehabilitation-intensive	Rehabilitation-intensive
Kuwait	High SDI	Rehabilitation-intensive	Rehabilitation-intensive	Rehabilitation-intensive	Rehabilitation-intensive	Rehabilitation-intensive	Rehabilitation-intensive	Rehabilitation-intensive
Kyrgyzstan	Low-middle SDI	Rehabilitation-intensive	Rehabilitation-intensive	Rehabilitation-intensive	Rehabilitation-intensive	Rehabilitation-intensive	Rehabilitation-intensive	Rehabilitation-intensive
Lao People's Democratic Republic	Low-middle SDI	Mortality-intensive	Mortality-intensive	Mortality-intensive	Mortality-intensive	Mixed	Mortality-intensive	Mortality-intensive
Latvia	High SDI	Mixed	Mixed	Mixed	Mixed	Mixed	Mixed	Prevention-intensive
Lebanon	High-middle SDI	Long-term-care-intensive	Long-term-care-intensive	Long-term-care-intensive	Long-term-care-intensive	Mixed	Long-term-care-intensive	Long-term-care-intensive
Lesotho	Low-middle SDI	Mixed	Mixed	Mixed	Mixed	Rehabilitation-intensive	Mixed	Mixed
Liberia	Low SDI	Mortality-intensive	Mortality-intensive	Mortality-intensive	Mortality-intensive	Mortality-intensive	Mortality-intensive	Mortality-intensive

Table S3. Country nursing priority profiles and sensitivity classifications

Country	SDI quintile	Primary profile	Age-standardized incidence	80+ sensitivity	P80 sensitivity	Number/Rate-only	Female model	Male model
Libya	High-middle SDI	Rehabilitation-intensive	Rehabilitation-intensive	Rehabilitation-intensive	Rehabilitation-intensive	Rehabilitation-intensive	Rehabilitation-intensive	Rehabilitation-intensive
Lithuania	High SDI	Prevention-intensive	Prevention-intensive	Prevention-intensive	Prevention-intensive	Mixed	Mixed	Prevention-intensive
Luxembourg	High SDI	Mixed	Mixed	Mixed	Mixed	Mixed	Mixed	Mixed
Madagascar	Low SDI	Mortality-intensive	Mortality-intensive	Mortality-intensive	Mortality-intensive	Mortality-intensive	Mortality-intensive	Mortality-intensive
Malawi	Low SDI	Mortality-intensive	Mortality-intensive	Mortality-intensive	Mortality-intensive	Mortality-intensive	Mortality-intensive	Mortality-intensive
Malaysia	High-middle SDI	Mixed	Mixed	Mixed	Mixed	Mixed	Mixed	Mixed
Maldives	Middle SDI	Mixed	Mixed	Mixed	Mixed	Mixed	Mixed	Mixed
Mali	Low SDI	Mortality-intensive	Mortality-intensive	Mortality-intensive	Mortality-intensive	Mortality-intensive	Mortality-intensive	Mortality-intensive
Malta	High-middle SDI	Mixed	Mixed	Mixed	Mixed	Mixed	Mixed	Mixed
Marshall Islands	Low-middle SDI	Mortality-intensive	Mortality-intensive	Mortality-intensive	Mortality-intensive	Mixed	Mortality-intensive	Mortality-intensive
Mauritania	Low-middle SDI	Mortality-intensive	Mortality-intensive	Mortality-intensive	Mortality-intensive	Mortality-intensive	Mortality-intensive	Mortality-intensive
Mauritius	High-middle SDI	Rehabilitation-intensive	Rehabilitation-intensive	Rehabilitation-intensive	Mixed	Rehabilitation-intensive	Rehabilitation-intensive	Mixed
Mexico	Middle SDI	Rehabilitation-intensive	Mixed	Rehabilitation-intensive	Rehabilitation-intensive	Mixed	Rehabilitation-intensive	Mixed
Micronesia (Federated States of)	Low-middle SDI	Mixed	Mixed	Mixed	Mixed	Mixed	Mixed	Mixed
Monaco	High SDI	Mixed	Mixed	Mixed	Mixed	Mixed	Mixed	Rehabilitation-intensive
Mongolia	Low-middle SDI	Rehabilitation-intensive	Rehabilitation-intensive	Rehabilitation-intensive	Rehabilitation-intensive	Rehabilitation-intensive	Rehabilitation-intensive	Rehabilitation-intensive
Montenegro	High-middle SDI	Prevention-intensive	Prevention-intensive	Prevention-intensive	Mixed	Mixed	Prevention-intensive	Prevention-intensive
Morocco	Low-middle SDI	Mortality-intensive	Mortality-intensive	Mortality-intensive	Mortality-intensive	Mixed	Mortality-intensive	Mortality-intensive
Mozambique	Low SDI	Mixed	Mixed	Mortality-intensive	Long-term-care-intensive	Mortality-intensive	Mixed	Mortality-intensive
Myanmar	Low-middle SDI	Mortality-intensive	Mortality-intensive	Mortality-intensive	Mortality-intensive	Mixed	Mortality-intensive	Mixed
Namibia	Low-middle SDI	Mixed	Mixed	Mixed	Mixed	Rehabilitation-intensive	Mixed	Rehabilitation-intensive
Nauru	Middle SDI	Mixed	Mixed	Mixed	Mixed	Mixed	Mortality-intensive	Mixed
Nepal	Low SDI	Mixed	Mixed	Mixed	Mixed	Rehabilitation-intensive	Mixed	Mixed
Netherlands	High SDI	Mixed	Mixed	Mixed	Mixed	Mixed	Mixed	Mixed
New Zealand	High SDI	Mixed	Mixed	Mixed	Mixed	Mixed	Mixed	Mixed
Nicaragua	Low-middle SDI	Rehabilitation-intensive	Rehabilitation-intensive	Rehabilitation-intensive	Mixed	Mixed	Rehabilitation-intensive	Mixed

Table S3. Country nursing priority profiles and sensitivity classifications

Country	SDI quintile	Primary profile	Age-standardized incidence	80+ sensitivity	P80 sensitivity	Number/Rate-only	Female model	Male model
Niger	Low SDI	Mortality-intensive	Mortality-intensive	Mortality-intensive	Mortality-intensive	Mortality-intensive	Mortality-intensive	Mortality-intensive
Nigeria	Low-middle SDI	Mortality-intensive	Mortality-intensive	Mixed	Mixed	Mixed	Mixed	Mixed
Niue	High-middle SDI	Mixed	Mixed	Mixed	Mixed	Mixed	Long-term-care-intensive	Mixed
North Macedonia	High-middle SDI	Mixed	Mixed	Mixed	Mixed	Mixed	Mixed	Mixed
Northern Mariana Islands	High-middle SDI	Mixed	Mixed	Mixed	Mixed	Mixed	Rehabilitation-intensive	Mixed
Norway	High SDI	Mixed	Mixed	Mixed	Mixed	Mixed	Mixed	Mixed
Oman	High-middle SDI	Mixed	Mixed	Mixed	Mixed	Mixed	Mixed	Mixed
Pakistan	Low-middle SDI	Mortality-intensive	Mortality-intensive	Mortality-intensive	Mortality-intensive	Mortality-intensive	Mortality-intensive	Mortality-intensive
Palau	High-middle SDI	Mixed	Mixed	Mixed	Mixed	Long-term-care-intensive	Mortality-intensive	Mixed
Palestine	Middle SDI	Mixed	Mixed	Mixed	Mixed	Mixed	Mixed	Mixed
Panama	Middle SDI	Rehabilitation-intensive	Rehabilitation-intensive	Rehabilitation-intensive	Rehabilitation-intensive	Rehabilitation-intensive	Rehabilitation-intensive	Rehabilitation-intensive
Papua New Guinea	Low SDI	Mortality-intensive	Mortality-intensive	Mortality-intensive	Mortality-intensive	Mixed	Mortality-intensive	Mortality-intensive
Paraguay	Middle SDI	Mixed	Mixed	Mixed	Mixed	Mixed	Mixed	Rehabilitation-intensive
Peru	Middle SDI	Rehabilitation-intensive	Rehabilitation-intensive	Rehabilitation-intensive	Rehabilitation-intensive	Rehabilitation-intensive	Mixed	Rehabilitation-intensive
Philippines	Middle SDI	Mixed	Mixed	Mixed	Mixed	Mixed	Mixed	Mixed
Poland	High-middle SDI	Prevention-intensive	Prevention-intensive	Prevention-intensive	Prevention-intensive	Mixed	Mixed	Prevention-intensive
Portugal	High-middle SDI	Mixed	Mixed	Mixed	Mixed	Mixed	Mixed	Mixed
Puerto Rico	High SDI	Long-term-care-intensive	Long-term-care-intensive	Long-term-care-intensive	Mixed	Mixed	Long-term-care-intensive	Long-term-care-intensive
Qatar	High SDI	Rehabilitation-intensive	Rehabilitation-intensive	Rehabilitation-intensive	Rehabilitation-intensive	Rehabilitation-intensive	Rehabilitation-intensive	Rehabilitation-intensive
Republic of Korea	High SDI	Prevention-intensive	Prevention-intensive	Prevention-intensive	Prevention-intensive	Mixed	Long-term-care-intensive	Prevention-intensive
Republic of Moldova	High-middle SDI	Rehabilitation-intensive	Rehabilitation-intensive	Rehabilitation-intensive	Rehabilitation-intensive	Rehabilitation-intensive	Rehabilitation-intensive	Mixed
Romania	High-middle SDI	Rehabilitation-intensive	Rehabilitation-intensive	Rehabilitation-intensive	Mixed	Mixed	Rehabilitation-intensive	Prevention-intensive
Russian Federation	High-middle SDI	Rehabilitation-intensive	Mixed	Rehabilitation-intensive	Rehabilitation-intensive	Mixed	Rehabilitation-intensive	Rehabilitation-intensive
Rwanda	Low SDI	Mortality-intensive	Mortality-intensive	Mortality-intensive	Mortality-intensive	Mortality-intensive	Mortality-intensive	Mortality-intensive
Saint Kitts and Nevis	High-middle SDI	Mixed	Mixed	Mixed	Mixed	Mixed	Long-term-care-intensive	Mixed

Table S3. Country nursing priority profiles and sensitivity classifications

Country	SDI quintile	Primary profile	Age-standardized incidence	80+ sensitivity	P80 sensitivity	Number/Rate-only	Female model	Male model
Saint Lucia	Middle SDI	Mixed	Mixed	Mixed	Mixed	Mixed	Mixed	Long-term-care-intensive
Saint Vincent and the Grenadines	Middle SDI	Mixed	Mixed	Mixed	Mixed	Mixed	Rehabilitation-intensive	Long-term-care-intensive
Samoa	Low-middle SDI	Mixed	Mixed	Mixed	Mixed	Mixed	Mixed	Rehabilitation-intensive
San Marino	High SDI	Mixed	Mixed	Mixed	Mixed	Mixed	Mixed	Mixed
Sao Tome and Principe	Low-middle SDI	Mortality-intensive	Mortality-intensive	Mortality-intensive	Mortality-intensive	Mortality-intensive	Long-term-care-intensive	Mortality-intensive
Saudi Arabia	High SDI	Rehabilitation-intensive	Rehabilitation-intensive	Rehabilitation-intensive	Mixed	Mixed	Mixed	Rehabilitation-intensive
Senegal	Low SDI	Mixed	Mixed	Mortality-intensive	Mortality-intensive	Mortality-intensive	Mixed	Mixed
Serbia	High-middle SDI	Mixed	Mixed	Mixed	Mixed	Mixed	Mixed	Mixed
Seychelles	High-middle SDI	Rehabilitation-intensive	Rehabilitation-intensive	Rehabilitation-intensive	Rehabilitation-intensive	Rehabilitation-intensive	Rehabilitation-intensive	Rehabilitation-intensive
Sierra Leone	Low SDI	Mixed	Mixed	Mixed	Mortality-intensive	Mortality-intensive	Mixed	Mortality-intensive
Singapore	High SDI	Rehabilitation-intensive	Mixed	Rehabilitation-intensive	Rehabilitation-intensive	Mixed	Rehabilitation-intensive	Rehabilitation-intensive
Slovakia	High-middle SDI	Prevention-intensive	Prevention-intensive	Prevention-intensive	Prevention-intensive	Mixed	Prevention-intensive	Prevention-intensive
Slovenia	High SDI	Mixed	Mixed	Mixed	Mixed	Mixed	Mixed	Mixed
Solomon Islands	Low SDI	Mortality-intensive	Mortality-intensive	Mortality-intensive	Mortality-intensive	Mixed	Mortality-intensive	Mortality-intensive
Somalia	Low SDI	Mortality-intensive	Mortality-intensive	Mortality-intensive	Mixed	Mortality-intensive	Mixed	Mortality-intensive
South Africa	Middle SDI	Rehabilitation-intensive	Rehabilitation-intensive	Rehabilitation-intensive	Rehabilitation-intensive	Rehabilitation-intensive	Rehabilitation-intensive	Rehabilitation-intensive
South Sudan	Low SDI	Mortality-intensive	Mortality-intensive	Mortality-intensive	Mortality-intensive	Mortality-intensive	Mortality-intensive	Mortality-intensive
Spain	High-middle SDI	Mixed	Mixed	Mixed	Mixed	Mixed	Mixed	Mixed
Sri Lanka	Middle SDI	Mixed	Mixed	Mixed	Mixed	Mixed	Mixed	Mixed
Sudan	Low-middle SDI	Mixed	Mixed	Mixed	Mixed	Rehabilitation-intensive	Mixed	Mixed
Suriname	Middle SDI	Mixed	Mixed	Mixed	Mixed	Mixed	Mixed	Mixed
Sweden	High SDI	Mixed	Mixed	Mixed	Mixed	Mixed	Mixed	Mixed
Switzerland	High SDI	Mixed	Mixed	Mixed	Mixed	Mixed	Mixed	Mixed
Syrian Arab Republic	Middle SDI	Rehabilitation-intensive	Rehabilitation-intensive	Rehabilitation-intensive	Rehabilitation-intensive	Rehabilitation-intensive	Rehabilitation-intensive	Rehabilitation-intensive
Taiwan	High SDI	Mixed	Mixed	Mixed	Mixed	Mixed	Mixed	Mixed
Tajikistan	Low-middle SDI	Rehabilitation-intensive	Rehabilitation-intensive	Rehabilitation-intensive	Rehabilitation-intensive	Rehabilitation-intensive	Rehabilitation-intensive	Rehabilitation-intensive

Table S3. Country nursing priority profiles and sensitivity classifications

Country	SDI quintile	Primary profile	Age-standardized incidence	80+ sensitivity	P80 sensitivity	Number/Rate-only	Female model	Male model
Thailand	Middle SDI	Rehabilitation-intensive	Rehabilitation-intensive	Rehabilitation-intensive	Mixed	Mixed	Mixed	Rehabilitation-intensive
Timor-Leste	Low SDI	Mortality-intensive	Mortality-intensive	Mortality-intensive	Mixed	Mortality-intensive	Mortality-intensive	Mortality-intensive
Togo	Low SDI	Mortality-intensive	Mortality-intensive	Mortality-intensive	Mortality-intensive	Mortality-intensive	Mortality-intensive	Mortality-intensive
Tokelau	Middle SDI	Long-term-care-intensive	Long-term-care-intensive	Mixed	Mixed	Mortality-intensive	Mortality-intensive	Long-term-care-intensive
Tonga	Middle SDI	Mixed	Mixed	Mixed	Mixed	Mixed	Mixed	Rehabilitation-intensive
Trinidad and Tobago	High-middle SDI	Mixed	Mixed	Mixed	Mixed	Mixed	Mixed	Mixed
Tunisia	Middle SDI	Long-term-care-intensive	Mixed	Long-term-care-intensive	Mixed	Mixed	Long-term-care-intensive	Mixed
Turkmenistan	Middle SDI	Rehabilitation-intensive	Rehabilitation-intensive	Rehabilitation-intensive	Rehabilitation-intensive	Rehabilitation-intensive	Rehabilitation-intensive	Rehabilitation-intensive
Tuvalu	Low-middle SDI	Mixed	Mixed	Mixed	Mixed	Rehabilitation-intensive	Mixed	Mixed
Türkiye	High-middle SDI	Mixed	Mixed	Mixed	Mixed	Mixed	Mixed	Mixed
Uganda	Low SDI	Mortality-intensive	Mortality-intensive	Mortality-intensive	Mortality-intensive	Mortality-intensive	Mortality-intensive	Mortality-intensive
Ukraine	High-middle SDI	Rehabilitation-intensive	Rehabilitation-intensive	Rehabilitation-intensive	Rehabilitation-intensive	Rehabilitation-intensive	Rehabilitation-intensive	Rehabilitation-intensive
United Arab Emirates	High SDI	Rehabilitation-intensive	Rehabilitation-intensive	Rehabilitation-intensive	Rehabilitation-intensive	Rehabilitation-intensive	Mixed	Rehabilitation-intensive
United Kingdom	High SDI	Mixed	Mixed	Mixed	Mixed	Mixed	Mixed	Mixed
United Republic of Tanzania	Low SDI	Mixed	Mixed	Mixed	Mixed	Mortality-intensive	Mortality-intensive	Mixed
United States Virgin Islands	High SDI	Mixed	Mixed	Mixed	Mixed	Mixed	Mixed	Mixed
United States of America	High SDI	Prevention-intensive	Prevention-intensive	Prevention-intensive	Prevention-intensive	Mixed	Prevention-intensive	Prevention-intensive
Uruguay	High-middle SDI	Long-term-care-intensive	Long-term-care-intensive	Long-term-care-intensive	Long-term-care-intensive	Mixed	Long-term-care-intensive	Long-term-care-intensive
Uzbekistan	Middle SDI	Rehabilitation-intensive	Rehabilitation-intensive	Rehabilitation-intensive	Rehabilitation-intensive	Rehabilitation-intensive	Rehabilitation-intensive	Rehabilitation-intensive
Vanuatu	Low-middle SDI	Mixed	Mixed	Mixed	Mixed	Rehabilitation-intensive	Mixed	Mixed
Venezuela (Bolivarian Republic of)	Low-middle SDI	Rehabilitation-intensive	Rehabilitation-intensive	Rehabilitation-intensive	Rehabilitation-intensive	Rehabilitation-intensive	Rehabilitation-intensive	Rehabilitation-intensive
Viet Nam	Middle SDI	Mixed	Long-term-care-intensive	Mixed	Mixed	Mixed	Mixed	Prevention-intensive
Yemen	Low SDI	Mortality-intensive	Mortality-intensive	Mortality-intensive	Mixed	Mixed	Mixed	Mortality-intensive
Zambia	Low-middle SDI	Mortality-intensive	Mortality-intensive	Mortality-intensive	Mortality-intensive	Mortality-intensive	Mortality-intensive	Mortality-intensive
Zimbabwe	Low-middle SDI	Mixed	Mixed	Mixed	Mixed	Mixed	Mortality-intensive	Mixed

Table S3. Country nursing priority profiles and sensitivity classifications

Country	SDI quintile	Primary profile	Age-standardized incidence	80+ sensitivity	P80 sensitivity	Number/Rate-only	Female model	Male model

Primary profile uses the prespecified P75 rule and 85+ share of YLDs as the long-term-care indicator. Age-standardized incidence sensitivity replaces the prevention-domain indicator with the GBD age-standardized incidence rate for falls. Sensitivity models correspond to the analyses shown in Figures S2-S5.

Table S4. Global age-sex stratified indicators in 2023

Age group	Sex	Incidence rate	YLD rate	YLDs per 1,000 falls	Deaths per 100,000 falls	YLD share of DALYs (%)
60-64 years	Both	4,795.8	1,319.8	275.2	296.3	76.3
60-64 years	Female	5,135.1	1,172.1	228.3	178.6	81.6
60-64 years	Male	4,437.8	1,475.5	332.5	439.9	72.4
65-69 years	Both	4,920.2	1,561.9	317.4	413.9	76.0
65-69 years	Female	5,607.3	1,462.0	260.7	276.4	79.5
65-69 years	Male	4,174.4	1,670.3	400.1	614.3	72.8
70-74 years	Both	5,834.6	1,825.9	312.9	581.8	72.9
70-74 years	Female	6,877.4	1,810.2	263.2	427.6	75.5
70-74 years	Male	4,644.8	1,843.9	397.0	842.3	70.2
75-79 years	Both	7,532.7	2,178.7	289.2	807.1	69.1
75-79 years	Female	9,074.5	2,294.9	252.9	599.2	72.5
75-79 years	Male	5,674.3	2,038.7	359.3	1,207.8	65.0
80-84 years	Both	10,547.9	2,680.1	254.1	1,110.3	64.8
80-84 years	Female	12,605.1	2,938.5	233.1	864.0	68.5
80-84 years	Male	7,764.7	2,330.5	300.1	1,651.4	59.3
85-89 years	Both	14,867.2	3,330.7	224.0	1,601.2	58.6
85-89 years	Female	17,436.0	3,719.8	213.3	1,285.7	62.7
85-89 years	Male	10,785.8	2,712.3	251.5	2,411.4	51.3
90-94 years	Both	18,939.7	4,002.1	211.3	2,373.7	50.8
90-94 years	Female	22,202.2	4,500.1	202.7	1,919.9	55.1
90-94 years	Male	12,701.6	3,049.9	240.1	3,890.3	41.7
95+ years	Both	21,648.0	4,468.7	206.4	3,167.1	44.7
95+ years	Female	25,272.9	5,001.1	197.9	2,692.4	47.7
95+ years	Male	12,706.5	3,155.5	248.3	5,496.0	35.9

Indicators are aggregated globally from the country-level master dataset for adults aged 60 years or older in 2023. Rates are crude rates per 100,000 persons.

Table S5. Oldest-old care pressure indicators in 2023

Group	80+ share of YLDs (%)	85+ share of YLDs (%)	90+ share of YLDs (%)	80+ share of DALYs (%)	85+ share of DALYs (%)	90+ share of DALYs (%)
Global	24.6	13.0	5.1	29.0	16.6	7.2
High SDI	31.6	17.8	7.5	37.6	23.0	10.9
High-middle SDI	20.4	10.4	3.7	25.3	14.2	5.7
Middle SDI	22.5	12.1	5.1	26.1	14.9	6.8
Low-middle SDI	20.3	9.1	2.8	23.6	11.7	4.2
Low SDI	24.1	12.8	5.1	28.4	16.4	7.0

Shares are calculated within adults aged 60 years or older in 2023. Global values are aggregated from all 204 countries and territories.

Table S6. Age-standardized risk-attributable YLD rates in 2023

Group	Sex	High alcohol use	Low bone mineral density	Occupational injuries	Smoking
Global	Both	17.4	138.3	97.5	6.8
Global	Female	6.3	163.0	52.8	3.6
Global	Male	28.6	108.9	141.9	10.3
High SDI	Both	30.7	169.6	103.7	8.7
High SDI	Female	14.5	199.2	64.9	5.9
High SDI	Male	46.7	133.9	141.3	11.8
High-middle SDI	Both	17.2	134.5	105.1	7.4
High-middle SDI	Female	2.9	151.7	52.9	2.0
High-middle SDI	Male	31.6	114.7	157.4	13.3
Middle SDI	Both	9.8	98.9	83.0	5.9
Middle SDI	Female	1.2	113.7	45.6	1.3
Middle SDI	Male	18.4	79.8	120.0	11.2
Low-middle SDI	Both	4.7	99.4	98.1	3.8
Low-middle SDI	Female	0.6	126.8	30.1	2.0
Low-middle SDI	Male	8.8	67.3	166.4	5.6
Low SDI	Both	2.6	65.6	90.1	2.5
Low SDI	Female	0.7	87.3	56.6	1.6
Low SDI	Male	4.5	42.2	124.2	3.4

Values are age-standardized YLD rates per 100,000 population in 2023. Risk attribution results are presented for the four prespecified risks only.
