# Supplementary material for: Country‐Level Burden Profiles for Fall‐Related Nursing Service Planning in Older Adults
Source: J Nurs Manag. 2026 Aug 2;2026:4990192. doi: 10.1155/jonm/4990192 (PMC13430041; doi:10.1155/jonm/4990192)
Supplement: Supplementary file 2 — Supporting Information 2 Reporting Guideline Checklist STROBE. [file JONM-2026-4990192-s001.docx]

**Reporting Guideline Checklist (STROBE)**

This checklist maps the main STROBE reporting domains to the current manuscript structure for an ecological, cross-national observational analysis.

| Checklist item | Status | Location in manuscript |
| --- | --- | --- |
| Title and abstract | Reported | Pages 1-2 |
| Background and rationale | Reported | Pages 2-3 |
| Objectives | Reported | Pages 2-3 |
| Study design | Reported | Pages 3-4 |
| Setting | Reported | Pages 3-4 |
| Participants / unit of analysis | Reported | Pages 4-5 |
| Variables and outcomes | Reported | Pages 4-5 |
| Data sources / measurement | Reported | Pages 3-5 |
| Bias / limitations of design | Partly reported | Pages 11-12 |
| Study size | Reported | Pages 3-4 |
| Statistical methods | Reported | Pages 5-6 |
| Descriptive data | Reported | Pages 6-9; Tables 1 and S1-S4 |
| Main results | Reported | Pages 6-9; Figures 1-3; Tables 1-2 |
| Other analyses | Reported | Pages 8-9; Figures S1-S8; Tables S2-S6 |
| Key results | Reported | Pages 9-10 |
| Limitations | Reported | Pages 11-12 |
| Interpretation | Reported | Pages 9-12 |
| Generalisability | Reported | Pages 11-12 |
| Funding | Reported | Title page |
